# Investigating the effects of absolute humidity and movement on COVID-19 seasonality in the United States

**DOI:** 10.1038/s41598-022-19898-8

**Published:** 2022-10-06

**Authors:** Gary Lin, Alisa Hamilton, Oliver Gatalo, Fardad Haghpanah, Takeru Igusa, Eili Klein

**Affiliations:** 1 Center for Disease Dynamics, Economics & Policy, 962 Wayne Avenue, Suite 530, Silver Spring, MD 20910-4433 USA; 2 Department of Civil and Systems Engineering, Johns Hopkins University, Baltimore, MD USA; 3 Department of Earth and Planetary Sciences, Johns Hopkins University, Baltimore, MD USA; 4 Center for Systems Science and Engineering, Johns Hopkins University, Baltimore, MD USA; 5 Department of Emergency Medicine, Johns Hopkins University, Baltimore, MD USA; 6 Department of Epidemiology, Johns Hopkins University, Baltimore, MD USA

**Keywords:** Infectious diseases, Epidemiology

## Abstract

Mounting evidence suggests the primary mode of SARS-CoV-2 transmission is aerosolized transmission from close contact with infected individuals. While transmission is a direct result of human encounters, falling humidity may enhance aerosolized transmission risks similar to other respiratory viruses (e.g., influenza). Using Google COVID-19 Community Mobility Reports, we assessed the relative effects of absolute humidity and changes in individual movement patterns on daily cases while accounting for regional differences in climatological regimes. Our results indicate that increasing humidity was associated with declining cases in the spring and summer of 2020, while decreasing humidity and increase in residential mobility during winter months likely caused increases in COVID-19 cases. The effects of humidity were generally greater in regions with lower humidity levels. Given the possibility that COVID-19 will be endemic, understanding the behavioral and environmental drivers of COVID-19 seasonality in the United States will be paramount as policymakers, healthcare systems, and researchers forecast and plan accordingly.

## Introduction

As of October 14, 2021, the coronavirus disease 2019 (COVID-19) pandemic has claimed over 720,000 lives in the United States alone, with more than 44.7 million confirmed cases^1^. Current evidence suggests that the primary mode of transmission of the severe acute respiratory syndrome coronavirus 2 (SARS-CoV-2) is close contact with infected individuals^2,3^. Aerosols^4,5^, which are particulates less than 5 µm in diameter^6,7^, likely play a significant role in transmission^8^. After the initial rise of cases in the early winter of 2020, cases remained severe through the spring before dropping in the summer. Given the shelter-in-place order in most states and the rise in humidity, cases generally decreased in May and stayed in lower ranges through the summer until the fall months. In most areas of the northern hemisphere, as fall turns to winter, the weather becomes colder and drier. Lower absolute humidity has been shown to be associated with increased transmission rates of other respiratory viruses (e.g., influenza)^9^, posing significant concerns regarding potential increases in the number of COVID-19 cases in the fall and winter. The surge in cases through the end of 2020 further supports the seasonal effects of COVID-19.

While several studies have suggested a relationship between climatic factors (e.g., temperature and/or humidity) and COVID-19^10–18^, the exact environmental and biological mechanism behind airborne and droplet transmission and viral survival of SARS-CoV-2^19^ is not yet clear. In influenza, lower atmospheric moisture has been shown to increase the production of aerosol nuclei and viral survival time^9^, which translates to higher risks of airborne and droplet transmission. Other climatic factors that may impact transmission include temperature and air quality^20,21^; nevertheless, absolute humidity can still provide a surrogate measure for indoor air moisture and temperature^22^.

Initial efforts to slow the spread of COVID-19 focused on reducing contacts between individuals through social-distancing measures such as large-scale lockdowns, which were significantly associated with reductions in cases^23^. However, as the initial lockdowns were lifted and the movement of individuals increased, the correlation between mobility and case growth rates weakened overall^24^, though upticks in cases were associated with increased mobility during national holidays^25^. During the months of 2020 and 2021 some counties and states saw increases in cases, while others observed decreases without corresponding increases in movement by any metric. Thus, other factors, including environmental factors, must also be considered as important transmission drivers.

Analyses of the factors influencing COVID-19 have used either climate data^21,26–28^ or human mobility data^23^, but no study to our knowledge has considered changes in both climate and human mobility on COVID-19 outbreaks in the United States. Preliminary studies have investigated these effects in China but did not consider varying sensitivities to humidity for different climatological regimes, leading to a weaker detection of humidity impacts on transmission risks in areas with higher variations of humidity^29^. Understanding the potential for climatic factors to increase transmission in the fall and winter is crucial for developing policies to combat the spread of SARS-CoV-2. While the interaction between environmental factors and human encounters is complex, accounting for this relationship is necessary for determining appropriate policies that will be effective at reducing transmissions. Furthermore, indoor gatherings typically increase in frequency and size in the winter and are one of the largest risk factors for transmission^7,30^. Therefore, greater understanding regarding the added risk of weather changes is needed to aid future decisions on restricting gatherings or implementing mandates for protective face coverings. In this study, we assessed the relative impact of absolute humidity and human mobility in different climatological regimes on reported cases of COVID-19 in the US.

## Results

### Partitioning climatological regimes

**Figure 1:**
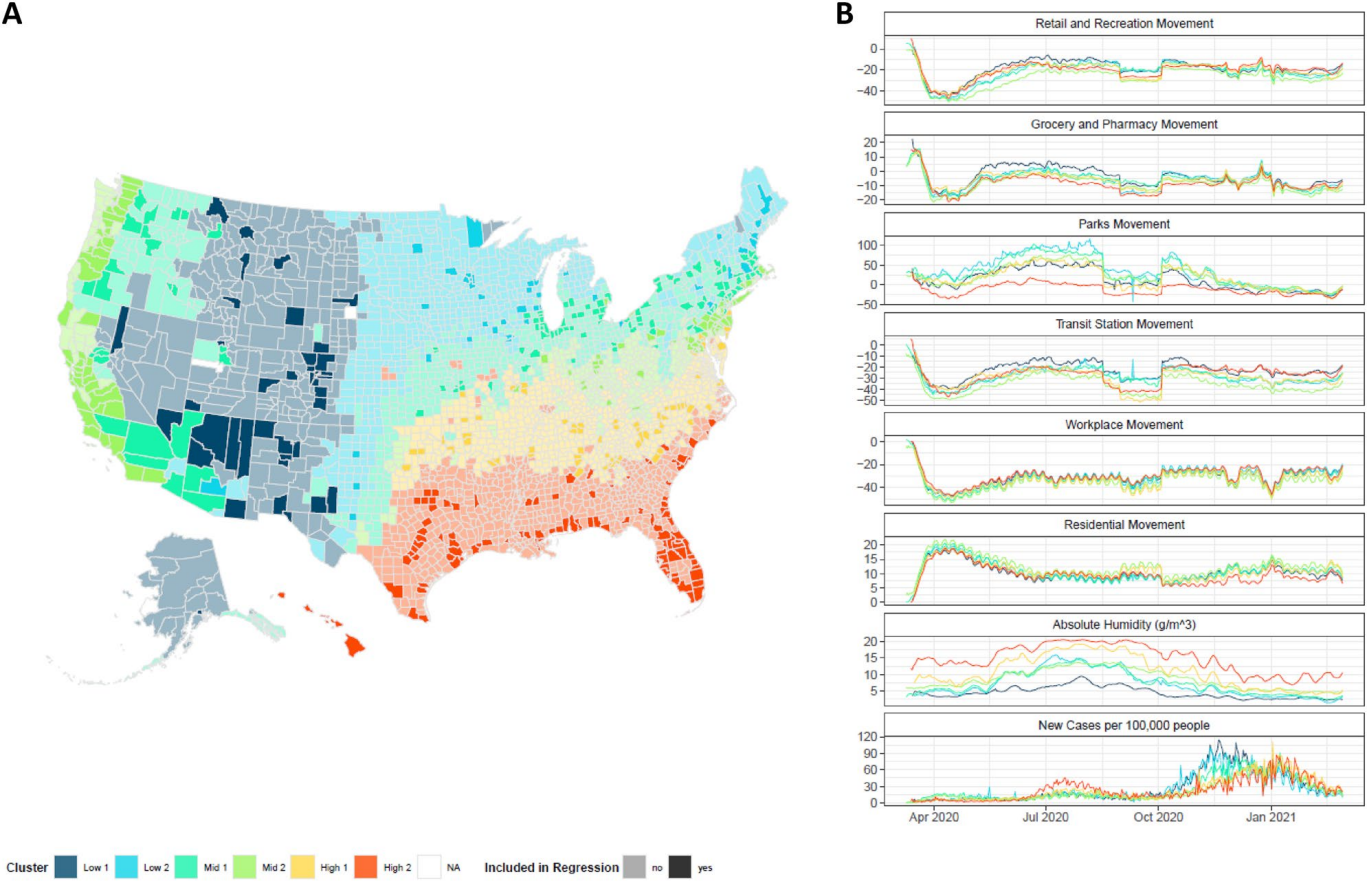
(**A**) Map of US Counties and their respective absolute humidity clusters. Each county is colored based on their cluster. Counties that are included in the regression analysis are indicated by a darker shade. The clustering analysis was conducted using a partitional algorithm that utilized dynamic time warping (DTW) to measure similarity between absolute humidity profiles of 3137 counties in the United States. Expectantly, the clustering of absolute humidity is related to the geography of the counties which serves as a proxy for regional weather patterns and different climatological regimes. (**B**) The cross-sectional smoothed mean of human encounter absolute humidity, and new case per 10,000 people trends for each cluster group of the 497 counties analyzed in the regression analysis. Map was generated using the ggplot package^31^ in R.

The US is geographically large and encompasses several different climatological regimes with varying absolute humidity trends. We partitioned all 3137 US counties into six exclusive clusters (Fig. 1) ranked by average absolute humidity (AH) using a dynamic time warping (DTW) algorithm which considers both magnitude and functional trends of AH (see “Methods”). The cluster with the lowest average AH was primarily located in the western region of the US, while the region with the highest average AH was located on the southern coast bordering the Gulf of Mexico. Large changes of humidity were seen in clusters High 1 and High 2 which, respectively, includes variances of 26.9 and 30.6 g/m^3^ (see Fig. S1), while Low 1 and Low 2 humidity clusters had a variance of 4.5 and 14.2 g/m^3^.

### Associations between humidity and cases rates

We conducted a regression on counties with more than 50,000 people using a generalized linear model (GLM) and controlling for individual movement and behavior with a metric from mobile phone data of visits to non-essential businesses (see Methods), we found that increases in AH were significantly negatively associated with cases per 100,000 of COVID-19 in all the non-high humidity regions (Table 1). We found that counties that belong to the least humid clusters, Low 1 and Low 2, had a 1 g/m^3^ increase in AH was associated with an average decrease of 14 percent reduction in cases over the entire duration, while the most humid clusters (High 1 and High 2) had a decrease of 4 percent in cases. The largest associations were seen in counties predominantly in the Rocky Mountains (Low 1; 20% decrease in daily cases), Upper Midwest/Northwest (Mid 1; 12% decrease in daily cases), West Coast/Texas/Northeast (Mid 2; 16% decrease in daily cases), and a region stretching along the western edge of the Midwest down to Texas (Low 2; 8% decrease in daily cases). Small but significant effects were detected in two high humidity clusters, both located in the southern region of the US (High 1 and High 2), with respective reductions of 6% and 1% in daily cases with a 1 g/m^3^ increase in AH.

**Table 1:** Untransformed GLM coefficient estimates for the entire study period.

Predictors	Low 1	Low 2	Mid 1	Mid 2	High 1	High 2
	Log-Mean	Log-Mean	Log-Mean	Log-Mean	Log-Mean	Log-Mean
Intercept	4.379*** (4.364–4.395)	3.439*** (3.423–3.455)	3.735*** (3.730–3.740)	3.885*** (3.876–3.894)	4.381*** (4.291–4.469)	3.270*** (3.254–3.285)
Absolute Humidity (14-day Lag)	− 0.221*** (− 0.223 to − 0.219)	− 0.084*** (− 0.085 to − 0.082)	− 0.123*** (− 0.124 to − 0.123)	− 0.171*** (− 0.171 to − 0.170)	− 0.060*** (− 0.060 to − 0.059)	− 0.015*** (− 0.015 to − 0.015)
Retail and Recreation (14-day Lag)	0.826*** (0.815–0.837)	0.839*** (0.829–0.850)	0.925*** (0.920–0.930)	0.515*** (0.511–0.519)	0.950*** (0.941–0.959)	1.299*** (1.293–1.305)
Grocery Stores and Pharmacies (14-day Lag)	− 0.354*** (− 0.361 to − 0.348)	− 0.145*** (− 0.152 to − 0.138)	0.040*** (0.037–0.042)	0.171*** (0.169–0.174)	− 0.223*** (− 0.228 to − 0.217)	− 0.130*** (− 0.134 to − 0.126)
Parks (14-day Lag)	− 0.536*** (− 0.543 to − 0.530)	− 0.123*** (− 0.128 to − 0.118)	− 0.156*** (− 0.159 to − 0.154)	− 0.200*** (− 0.202 to − 0.198)	− 0.379*** (− 0.383 to − 0.374)	− 0.984*** (− 0.988 to − 0.979)
Transit Stations (14-day Lag)	− 0.134*** (− 0.143 to − 0.125)	− 0.519*** (− 0.528 to − 0.511)	− 0.762*** (− 0.766 to − 0.758)	− 0.602*** (− 0.607 to − 0.598)	− 0.350*** (− 0.356 to − 0.344)	− 0.339*** (− 0.343 to − 0.335)
Workplaces (14-day Lag)	− 0.592*** (− 0.599 to − 0.585)	− 0.560*** (− 0.569 to − 0.552)	− 0.386*** (− 0.390 to − 0.383)	− 0.683*** (− 0.686 to − 0.680)	− 0.762*** (− 0.767 to − 0.757)	− 0.541*** (− 0.544 to − 0.538)
Residential (14-day Lag)	− 0.601*** (− 0.611 to − 0.591)	− 0.425*** (− 0.437 to − 0.413)	− 0.166*** (− 0.171 to − 0.161)	− 0.576*** (− 0.580 to − 0.572)	− 0.583*** (− 0.591 to − 0.575)	− 0.269*** (− 0.273 to − 0.265)
Observations	9557	7987	25,568	27,087	16,581	25,916

Untransformed coefficient (*β*) estimates for GLM Regression against new cases per 100,000 from March 10, 2020 to March 1, 2021. The 95% confidence intervals are shown in parenthesis. Estimated coefficients for county-level fixed effects and epidemiological terms (immunity factor and lagged daily cases) are not shown.

*p < 0.05 **p < 0.01 ***p < 0.001.

The overall associations between AH and COVID-19 cases were negatively correlated when disaggregated across the time periods (Tables 2 and 3). The regression showed that AH had strong associations in the Mid 2 cluster, located in West Coast/Texas/Northeast, during the spring and summer months of 2020 (Table 2). In the fall of 2020 and spring of 2021, AH associations were generally stronger in counties from Mid 2 and High 1 clusters, which are in the West Coast, Texas, Northeast and Southern regions of the US (Table 3).

**Table 2:** Untransformed GLM coefficient estimates for the 2020 spring to fall period.

Predictors	Low 1	Low 2	Mid 1	Mid 2	High 1	High 2
	Log-Mean	Log-Mean	Log-Mean	Log-Mean	Log-Mean	Log-Mean
Intercept	2.064*** (1.982–2.145)	2.198*** (2.159–2.236)	3.064*** (3.053–3.074)	3.033*** (3.014–3.053)	1.020*** (0.952–1.087)	− 2.835*** (− 2.875 to − 2.795)
Absolute Humidity (14-day Lag)	− 0.069*** (− 0.073 to − 0.065)	− 0.038*** (− 0.041 to − 0.035)	− 0.098*** (− 0.099 to − 0.097)	− 0.108*** (− 0.109 to − 0.106)	0.071*** (0.069–0.073)	0.221*** (0.220–0.222)
Retail and Recreation (14-day Lag)	1.313*** (1.279–1.348)	0.276*** (0.247–0.305)	0.709*** (0.700–0.719)	0.288*** (0.280–0.296)	0.866*** (0.847–0.884)	0.537*** (0.523–0.550)
Grocery Stores and Pharmacies (14-day Lag)	− 0.148*** (− 0.166 to − 0.130)	0.096*** (0.079–0.113)	0.352*** (0.347–0.357)	0.492*** (0.487–0.496)	− 0.112*** (− 0.125 to − 0.098)	0.261*** (0.253–0.269)
Parks (14-day Lag)	− 0.545*** (− 0.567 to − 0.523)	0.098*** (0.085–0.111)	− 0.184*** (− 0.190 to − 0.179)	− 0.239*** (− 0.244 to − 0.235)	− 0.144*** (− 0.153 to − 0.136)	0.528*** (0.514–0.541)
Transit Stations (14-day Lag)	− 0.463*** (− 0.497 to − 0.430)	− 1.021*** (− 1.052 to − 0.989)	− 0.633*** (− 0.645 to − 0.621)	− 0.589*** (− 0.603 to − 0.576)	− 0.230*** (− 0.247 to − 0.213)	− 0.411*** (− 0.423 to − 0.400)
Workplaces (14-day Lag)	− 0.650*** (− 0.669 to − 0.631)	− 0.544*** (− 0.574 to − 0.514)	− 0.696*** (− 0.705 to − 0.687)	− 0.900*** (− 0.909 to − 0.892)	− 0.644*** (− 0.663 to − 0.625)	− 0.072*** (− 0.083 to − 0.061)
Residential (14-day Lag)	− 0.256*** (− 0.278 to − 0.233)	− 0.579*** (− 0.615 to − 0.543)	− 0.356*** (− 0.367 to − 0.345)	− 0.782*** (− 0.791 to − 0.773)	− 0.233*** (− 0.256 to − 0.209)	0.176*** (0.165–0.188)
Observations	3604	2903	9781	11,260	6446	11,460

Untransformed coefficient (*β*) estimates for GLM Regression against new cases per 100,000 from March 10, 2020 to September 30, 2021. The 95% confidence intervals are shown in parenthesis. Estimated coefficients for county-level fixed effects and epidemiological terms (immunity factor and lagged daily cases) are not shown.

*p < 0.05 **p < 0.01 ***p < 0.001.

**Table 3:** Untransformed GLM coefficient estimates for the 2020 winter and 2021 spring seasons.

Predictors	Low 1	Low 2	Mid 1	Mid 2	High 1	High 2
	Log-Mean	Log-Mean	Log-Mean	Log-Mean	Log-Mean	Log-Mean
Intercept	5.411*** (5.391–5.431)	6.039*** (6.013–6.066)	5.782*** (5.772–5.791)	4.553*** (4.540–4.566)	6.410*** (6.318–6.498)	5.188*** (5.168–5.207)
Absolute Humidity (14-day Lag)	− 0.141*** (− 0.144 to − 0.138)	− 0.093*** (− 0.096 to − 0.091)	− 0.151*** (− 0.152 to − 0.150)	− 0.220*** (− 0.221 to − 0.219)	− 0.159*** (− 0.160 to − 0.157)	− 0.093*** (− 0.093 to − 0.092)
Retail and Recreation (14-day Lag)	0.329*** (0.314–0.344)	0.780*** (0.764–0.795)	0.567*** (0.559–0.575)	− 0.167*** (− 0.175 to − 0.158)	0.450*** (0.436–0.464)	0.511*** (0.501–0.521)
Grocery Stores and Pharmacies (14-day Lag)	0.312*** (0.302–0.322)	0.380*** (0.369–0.391)	0.501*** (0.497–0.506)	0.782*** (0.777–0.788)	0.200*** (0.191–0.209)	0.367*** (0.359–0.374)
Parks (14-day Lag)	− 0.518*** (− 0.527 to − 0.509)	− 0.030*** (− 0.037 to − 0.022)	0.267*** (0.263–0.270)	0.277*** (0.274–0.281)	− 0.249*** (− 0.254 to − 0.243)	− 0.736*** (− 0.743 to − 0.729)
Transit Stations (14-day Lag)	0.244*** (0.230–0.257)	0.129*** (0.116–0.142)	− 0.171*** (− 0.178 to − 0.165)	− 0.023*** (− 0.027 to − 0.019)	0.079*** (0.068–0.089)	− 0.038*** (− 0.046 to − 0.031)
Workplaces (14-day Lag)	0.017*** (0.007–0.027)	0.469*** (0.458–0.480)	0.514*** (0.509–0.519)	0.347*** (0.342–0.352)	− 0.145*** (− 0.152 to − 0.138)	− 0.081*** (− 0.086 to − 0.077)
Residential (14-day Lag)	0.545*** (0.529–0.561)	1.322*** (1.304–1.340)	1.273*** (1.265–1.281)	0.931*** (0.923–0.938)	0.218*** (0.207–0.229)	0.223*** (0.216–0.230)
Observations	5953	5084	15,787	15,827	10,135	14,456

Untransformed coefficient (*β*) estimates for GLM Regression against new cases per 100,000 from October 1, 2020 to March 1, 2021. The 95% confidence intervals are shown in parenthesis. Estimated coefficients for county-level fixed effects and epidemiological terms (immunity factor and lagged daily cases) are not shown.

*p < 0.05 **p < 0.01 ***p < 0.001.

### Associations between movement and case rates

In general, movement effects on daily cases are larger than absolute humidity effects, with visits to retail and recreation positively associated with new COVID-19 cases in most of the clusters (Table 1). Mobility trends for retail & recreation and grocery stores & pharmacies had a larger positive effect during the earlier phase of the pandemic for most clusters (March 10 to September 30, 2020) compared to the later phase spanning from October 1, 2020 to March 1, 2021. The residential mobility trend was associated with a decrease in new cases in most clusters during the earlier phase of the pandemic (Table 2), while having a positive effect on daily cases during the later phase (Table 3).

### Detecting multicollinearity between movement and absolute humidity

To understand the collinearity of the combined regressions shown in Tables 1, 2 and 3, we conducted robustness checks with additional regressions that included the AH and the mobility trends separately (See Tables S1–S18). Additionally, we calculated the Generalized Variational Inflation Factor (GVIF) for the regressions in our robustness checks. Workplaces and Residential Mobility Trends were the least collinear with other independent variables (absolute humidity, immunity factor, and previous 14-day caseload) supported by GVIF values less than 2. Mobility trends in Retail and Recreation Areas and Grocery Stores and Pharmacies were mostly non-collinear with few exceptions with GVIF values ranging between with a mean of 1.53 (range: 1.15–2.30) and 1.65 (1.28–2.63). And finally, Transit Stations and Parks demonstrated the most collinearity with mean GVIF values of 2.15 (1.45–3.71) and 2.01 (1.56–2.83).

## Discussion

**Figure 2:**
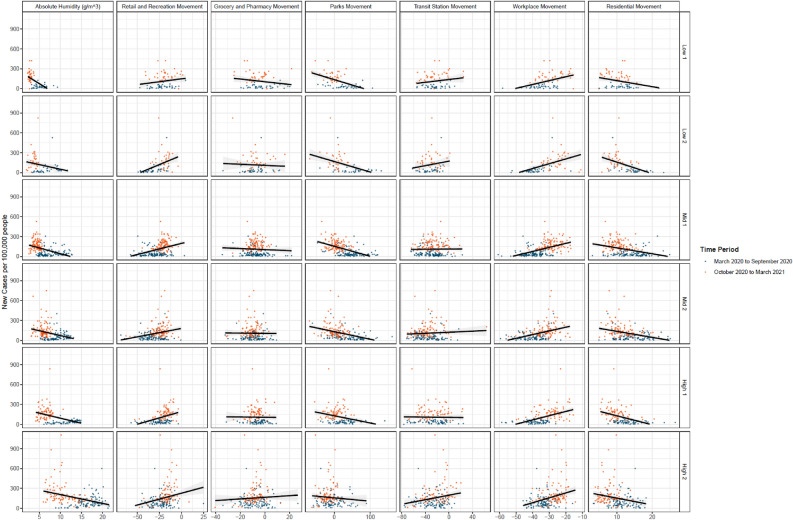
The average daily new cases per 100,000 people plotted against the average Google Mobility Measure of 497 counties for the entire study duration. The plots are organized by type of movement and cluster group. For each plot, we added a simple linear trend line with shaded standard errors.

As the COVID-19 epidemic continues in the US and given the surge of COVID-19 in the winter seasons, there is renewed interest in understanding the relationship between outbreaks and seasonal changes, especially climatological factors related to outdoor and indoor humidity. This is not the first study to investigate humidity impacts on transmission, which been associated with increased transmission of respiratory pathogens (e.g., influenza) and SARS-CoV-2. While SARS-CoV-2 is a novel human virus, other pandemic coronaviruses (e.g., MERS-CoV and SARS-CoV-1)^9,32–35^ have also been associated with increased transmission in the winter, thus suggesting similar implications for SARS-CoV-2. Here, we found that the relative effect of absolute humidity on transmissions has so far been significant and was greatest in the Western, upper Midwest, and Northeast regions of the United States, which were clustered into the driest climatological regimes. These results support the hypothesis that falling rates of absolute humidity magnify the transmission risk of SARS-CoV-2, particularly in regions that are more arid and dry^36^. This effect was less noticeable for more humid regions, such as the coastal and southern counties of the US (Fig. 2).

The effects of behavior and nonpharmaceutical interventions (NPI) are observed in our analysis when we disaggregate the analysis between the early and later phases of the pandemic. In the early phase of the pandemic, we see that an increase in mobility trends for retail & recreation resulted in an increase in daily cases, which measures visits to restaurants, cafes, shopping centers, theme parks, museums, libraries, and movie theaters. While in the later stages during the fall and winter of 2020, retail & recreation mobility had a lesser effect since many of those establishments were closed due to NPI policies. Furthermore, increases in residential mobility played a larger role in transmission, especially during the winter holidays when travel between residential homes occurred at a higher incidence.

The relationship between humidity and transmission is not fully clear, but several studies have shown that as absolute humidity decreases, survival times for enveloped viruses increase nonlinearly, including other coronaviruses^9,22,37,38^. Our findings support the hypothesis of a nonlinear relationship since the log-linear effects between humidity and case growth varied between climatological regimes. Our stratified regression and Fig. 2 show that different climatological regimes have different sensitivities to humidity changes. The increased survival of the virus in lower AH may be compounded by increased binding capacity, thereby enhancing the potential infectivity of the virus^39^. As AH falls, relative humidity indoors also decreases, which may increase susceptibility to airborne diseases^40^. This association suggests that increased humidification of indoor air in high transmission settings may help decrease the burden of COVID-19.

Given that our results suggest COVID-19 cases will increase significantly during winters, areas where humidity typically falls earlier in the fall (e.g., the upper Midwest) are likely to see cases increase earlier. In contrast, more humid regions (e.g., Gulf Coast areas) will likely observe outbreaks later in the winter. However, the results demonstrate that mobility had a larger and significant impact on cases, particularly when humidity was unchanging in the summer. Consequently, falling temperatures and holiday celebrations are likely to increase the risk of people gathering in indoor spaces for longer durations, resulting in a surge of COVID-19 cases through the winter, given that there are no substantial changes in population immunity and behavior.

The prior influenza pandemic in 2009 is instructive here, as increased contact patterns that occurred in the fall likely combined with falling humidity to drive transmission, which resulted in the peak of infections occurring significantly earlier than other years. Given the uncertainty and nonlinear effects of humidity on transmission, increasing vaccination, proper social distancing, and improving healthcare capacities can potentially reduce the toll of the COVID-19 pandemic. In addition, the uncertainty regarding the role of children in transmission^41–43^, who remain largely unvaccinated, suggests that proper precautions related to opening schools is warranted as the potential for transmission increases. While studies linking schools to outbreaks to date have been limited, few have occurred during the winter when transmission is higher.

We suspected that a relationship between human behavior and climate might exist which can cause variations in encounters. During winter months, the likelihood of being indoors increases especially in colder climates. To investigate this potential interaction, we conducted a collinear analysis. We can interpret this collinear analysis as residential and workplace movement patterns not being collinear with meteorological conditions (absolute humidity) and epidemiological factors (immunity factor and new cases per 100,000 (14-day Lag)). Retail/recreation and grocery/pharmacies are moderately collinear, while transit stations and parks were the most collinearly related to meteorological and epidemiological variables.

One limitation of this study includes changing social distancing dynamics and masking adherence between counties. We attempted to account for county-level heterogeneities using fixed effects for each county, but these are static effects. Furthermore, it is difficult to disentangle the epidemiological dynamics that cause exponential growth of cases. Events related to evacuation in natural disasters or mass-gatherings during the summer of 2020 that were not reflected in the Google Mobility Data^44^ may bias the analysis. Also, as with many COVID-19 analyses on retrospective data, the differences in testing rates at the county-level will result in varying detection rates of actual cases. Potential variations around vaccination efficacy for variants and within-host changes will impact the magnitude and exact timing of outbreaks^45^.

Transmission of SARS-CoV-2 will likely increase during the winters in the United States and other temperate regions in the northern hemisphere due in part to falling humidity. Studies of prior viruses and preliminary studies of the SARS-CoV-2 virus underpin the theoretical connection between humidity and transmission of droplet and aerosols. Nevertheless, mobility is still a significant driver of transmission.

## Methods

### Study design

The United States is geographically large, and the timing and magnitude of changes in absolute humidity can vary widely across regions. In order to account for regional differences in humidity, we utilized a partitional clustering algorithm with dynamic time warping (DTW) similarity measurements^46^ to classify the absolute humidity temporal profile for all observed counties into six exclusive clusters that are ranked based on average humidity. The clustering algorithm was implemented using the *dtw* package in R^47^. These clusters are ranked from lowest to highest as *Low 1, Low 2, Mid 1, Mid 2, High 1,* and *High 2.* Clustering allowed us to designate groups of counties based on temporal, climatological regimes and to stratify different absolute humidity patterns, which reduces group-level effects and enhances the independence of the data points. The DTW clustering of absolute humidity was conducted on a larger set of 3,137 counties. In the regression analysis, we included data from a subset of counties that had more than twenty cumulative confirmed cases and a population of more than 50,000 people. We excluded any days with fewer than 20 cumulative confirmed cases within each county because early transmission dynamics had a high rate of undetected cases^48^, making the data unreliable for this analysis. The final dataset used in the regression analysis included 497 counties, where separate panel data GLM was conducted on counties in each cluster (N_Low1_ = 39, N_Low 2_ = 42, N_Mid1_ = 118, N_Mid2_ = 108, N_High1_ = 78, and N_High2_ = 105). We assessed the results of the model over the entirety of the dataset and two time periods in 2020–2021: (1) the entire duration of the dataset (March 10, 2020 to March 1, 2021), (2) spring and summer when humidity increases (March 10, 2020 to September 30, 2020), and (3) the fall and winter months when humidity decreases to its lowest point (October 1, 2020 to March 1, 2021).

### Data sources

Confirmed case data were extracted from the Johns Hopkins Center for Systems Science and Engineering^1^ for each county. Population data were obtained from the US Census Bureau^49^ for 3,137 counties from March 10, 2020 to March 1, 2021. Daily cases were obtained from the confirmed case count by taking a simple difference between the days. Any data incongruencies, such as negative case counts, were omitted in our analysis.

Daily average absolute humidity for each US county, excluding territories, was calculated using temperature and dewpoint data from the National Centers for Environmental Information^50^ at the National Oceanic and Atmospheric Administration (NOAA). Time series data for the year 2020 from US weather stations were acquired from the NOAA Global Summary of the Day Index^51^. Weather stations were mapped using latitude and longitude to corresponding counties using the Federal Communications Commission (FCC) Census Block API^52^. For counties without a weather station, we used data from the nearest station, which was calculated based on distance from the county’s spatial centroid using the haversine formula. In cases where counties contained multiple stations, data were averaged across all stations in a county. Absolute humidity was calculated using average daily temperature and average daily dew point (see Alduchov and Eskridge^53^).

Data on mobility from March 10, 2020 to March 1, 2021 was obtained from the Google COVID-19 Community Mobility Reports^54^. We specifically utilized the metric that measures visits to grocery stores & pharmacies, parks, transit stations, retail & recreation, residential, and workplaces by comparing the median rate on the county-level to a 5-week period Jan 3–Feb 6, 2020. The measure was calculated as the percent difference from before policy interventions (e.g., shelter-in-place orders) began to impact movement. This temporal measure allowed us to compare movement differences across counties.

### Statistical analysis

For each humidity cluster that was classified using the DTW algorithm, we conducted three multivariate regressions using a generalized linear model (GLM) that assessed the time-weighted association between absolute humidity and non-essential visits with the number of new coronavirus cases (Eqs. 1–3). The GLM regression results in Tables 1, 2 and 3 are described in the following equation,

1 $$\begin{aligned} \log \left( {Y_{it} } \right) = & \log \left( N \right) + \alpha + \beta_{1} IM_{t} + \beta_{2} y_{{i\left( {t - \delta } \right)}} + \beta_{3} AH_{{i\left( {t - \delta } \right)}} + \beta_{4} RR_{{i\left( {t - \delta } \right)}} + \beta_{5} GP_{{i\left( {t - \delta } \right)}} \\ & + \beta_{6} PK_{{i\left( {t - \delta } \right)}} + \beta_{7} TS_{{i\left( {t - \delta } \right)}} + \beta_{8} WP_{{i\left( {t - \delta } \right)}} + \beta_{9} RD_{{i\left( {t - \delta } \right)}} + \gamma_{i} + \epsilon_{it} \\ \end{aligned}$$

where *Y*_*it*_, is the number of daily COVID-19 cases for county *i* at time *t*, log(*N*) is an offset term to control for population-size, and *α* is the intercept. In order to account for population immunity and exponential growth dynamics, we added the independent variables cumulative cases per 100,000, *IM*_*t*_, and lagged daily cases per 100,000, *y*_*i(t-δ)*_ to the regression models. Absolute humidity, *AH*_*i(t-δ)*_ is smoothed using a 7-day moving average and lagged by *δ* days. Google mobility trends to retail and recreation, *RR*_*i(t-δ)*_, grocery and pharmacies, *GP*_*i(t-δ)*_, parks, *PK*_*i(t-δ)*_, transit stations, *TS*_*i(t-δ)*_, workplaces, *WP*_*i(t-δ)*_, residential places, *RD*_*i(t-δ)*_, are smoothed using a 7-day moving average, lagged by *δ* days, and rescaled and centered on the mean. Fixed effects γ_i_ for each county were added to capture unobserved heterogeneities between counties. For our study, we assumed that the lag length *δ* was equal to 14 days, which is based on previous studies investigating lagged effects due to the incubation period of COVID-19^55^. As our outcome variable was daily cases, we modeled the variable as a Poisson distributed random variable with a log-transformed link function. Standard errors were calculated for the estimated linear coefficients *β*.

We conducted additional regressions on the absolute humidity and mobility measures as predictors individually to test for robustness. Specifically, we fit a GLM with absolute humidity for each humidity cluster and one measure from rescaled Google COVID-19 Community Mobility as linear predictors for new daily cases, as described in Eqs. (2) to (8).

2 $$\log \left( {Y_{it} } \right) = \log \left( N \right) + \alpha + \beta_{1} IM_{t} + \beta_{2} y_{{i\left( {t - \delta } \right)}} + \beta_{3} AH_{{i\left( {t - \delta } \right)}} + \gamma_{i} + \epsilon_{it}$$

3 $$\log \left( {Y_{it} } \right) = \log \left( N \right) + \alpha + \beta_{1} IM_{t} + \beta_{2} y_{{i\left( {t - \delta } \right)}} + \beta_{3} AH_{{i\left( {t - \delta } \right)}} + \beta_{4} RR_{{i\left( {t - \delta } \right)}} + \gamma_{i} + \epsilon_{it}$$

4 $$\log \left( {Y_{it} } \right) = \log \left( N \right) + \alpha + \beta_{1} IM_{t} + \beta_{2} y_{{i\left( {t - \delta } \right)}} + \beta_{3} AH_{{i\left( {t - \delta } \right)}} + \beta_{4} GP_{{i\left( {t - \delta } \right)}} + \gamma_{i} + \epsilon_{it}$$

5 $$\log \left( {Y_{it} } \right) = \log \left( N \right) + \alpha + \beta_{1} IM_{t} + \beta_{2} y_{{i\left( {t - \delta } \right)}} + \beta_{3} AH_{{i\left( {t - \delta } \right)}} + \beta_{4} PK_{{i\left( {t - \delta } \right)}} + \gamma_{i} + \epsilon_{it}$$

6 $$\log \left( {Y_{it} } \right) = \log \left( N \right) + \alpha + \beta_{1} IM_{t} + \beta_{2} y_{{i\left( {t - \delta } \right)}} + \beta_{3} AH_{{i\left( {t - \delta } \right)}} + \beta_{4} TS_{{i\left( {t - \delta } \right)}} + \gamma_{i} + \epsilon_{it}$$

7 $$\log \left( {Y_{it} } \right) = \log \left( N \right) + \alpha + \beta_{1} IM_{t} + \beta_{2} y_{{i\left( {t - \delta } \right)}} + \beta_{3} AH_{{i\left( {t - \delta } \right)}} + \beta_{4} RD_{{i\left( {t - \delta } \right)}} + \gamma_{i} + \epsilon_{it}$$

8 $$\log \left( {Y_{it} } \right) = \log \left( N \right) + \alpha + \beta_{1} IM_{t} + \beta_{2} y_{{i\left( {t - \delta } \right)}} + \beta_{3} AH_{{i\left( {t - \delta } \right)}} + \beta_{4} WP_{{i\left( {t - \delta } \right)}} + \gamma_{i} + \epsilon_{it}$$

To demonstrate robustness in the coefficient estimates, the coefficients in the combined regression analyses with absolute humidity and all mobility trends (Eq. (1)) were compared to the regression coefficients for absolute humidity and each mobility trend (Eqs. (2)–(8)). The analysis using GLM was conducted using the *stats* package in R (Version 4.0.2). All untransformed coefficient estimates are located in (Tables 1, 2 and 3). In the main text, we reported the logit-transformed estimates as relative change in cases per unit increase (1 g/m^3^) of absolute humidity. Given the log-linear relationship in a Poisson regression between the covariates and response variable, we can calculate the percent change in daily cases for a unit increase of a covariate to be equal to exp (*β*) − 1. For example, if β = − 0.112 for absolute humidity, we would state that there is a 9% (= exp (− 0.112) − 1) reduction for 1 g/m^3^ increase in absolute humidity. To verify that mulicollinearity is not a major issue, we conducted a collinearity analysis by calculating the Generalized Variational Inflation Factor (GVIF) for all regressions, which are listed in Table S19.

In addition to running a GLM regression, we also discretized the data based on months for each humidity cluster and calculated the Pearson correlation coefficient for absolute humidity and Google Mobility Trends against new cases (Fig. S2). Stationarity was checked for absolute humidity and Google mobility trends using the Levin-Lin-Chu unit-root test for unbalanced panel data for the three periods that were analyzed aforementioned regressions. Results for the stationarity are listed in Table S20 in the supplement.

We tested for robustness and externally validated our regressions by conducting additional analysis using K-folds cross-validation. We validated the coefficient estimation of all the GLMs mentioned previously by showing that the relative effect size for each regression was similar. The analysis was conducted over 100 folds or iterations with separate training and test sets derived from a subset of the county-level data. We used test sets for each fold where the mean square error (MSE) was calculated for each fit and shown in Table S22 in the supplement. In order to minimize overfitting, we also excluded county-level fixed effects in our cross-validation analysis. Additionally, we show the 95% confidence intervals of all parameter estimations using the GLM model that includes all variables in Table S23.

## Supplementary Information

Supplementary Information.

## Data Availability

The data that support the findings of this study are openly available through the Johns Hopkins Center for Systems Science and Engineering, Unacast Social Distancing Scorecard, and NOAA National Centers for Environmental Information. Population data can be found through the US Census Bureau Website. All input data and code used to conduct the analysis and generate figures are also available on Github at https://github.com/CDDEP-DC/COVID-Humidity-Mobility-GAM.
