# Supplementary material for: Investigating the effects of absolute humidity and movement on COVID-19 seasonality in the United States
Source: Sci Rep. 2022 Oct 6;12:16729. doi: 10.1038/s41598-022-19898-8 (PMC9537426; doi:10.1038/s41598-022-19898-8)
Supplement: Supplementary file 1 — Supplementary Information. [file 41598_2022_19898_MOESM1_ESM.docx]

Supplementary Materials for

**Investigating the effects of absolute humidity and movement on COVID-19 seasonality in the United States**

Gary Lin^1^, Alisa Hamilton^1^, Oliver Gatalo^1^, Fardad Haghpanah^1^, Takeru Igusa^2,3,4^, Eili Klein^1,5,6^, For the CDC MInD-Healthcare Network

1. Center for Disease Dynamics, Economics & Policy, Silver Spring, MD, USA
2. Department of Civil and Systems Engineering, Johns Hopkins University, Baltimore, MD, USA
3. Department of Earth and Planetary Sciences, Johns Hopkins University, Baltimore, MD, USA
4. Center for Systems Science and Engineering, Johns Hopkins University, Baltimore, MD, USA
5. Department of Emergency Medicine, Johns Hopkins University, Baltimore, MD, USA
6. Department of Epidemiology, Johns Hopkins University, Baltimore, MD, USA

| **Table S1.** GLM Regression against new cases for Low 1 cluster for the entire year. The 95% confidence intervals are shown in parenthesis. Estimated coefficients for county-level fixed effects and epidemiological terms (immunity factor and lagged daily cases) are not shown. | | | | | | | | |
| --- | --- | --- | --- | --- | --- | --- | --- | --- |
|  | **All**  **[Eq 1]** | **Absolute Humidity**  **[Eq 2]** | **Retail and Rec**  **[Eq 3]** | **Grocery and Pharmacy**  **[Eq 4]** | **Parks**  **[Eq 5]** | **Transit**  **[Eq 6]** | **Workplaces**  **[Eq 7]** | **Residential**  **[Eq 8]** |
| *Predictors* | *Log-Mean* | *Log-Mean* | *Log-Mean* | *Log-Mean* | *Log-Mean* | *Log-Mean* | *Log-Mean* | *Log-Mean* |
| Intercept | 4.379 ^***^ (4.364 – 4.395) | 3.904 ^***^ (3.889 – 3.919) | 4.197 ^***^ (4.181 – 4.212) | 3.965 ^***^ (3.950 – 3.981) | 3.886 ^***^ (3.870 – 3.901) | 4.049 ^***^ (4.034 – 4.065) | 3.992 ^***^ (3.977 – 4.007) | 4.136 ^***^ (4.121 – 4.151) |
| Absolute Humidity  (14-day Lag) | -0.221 ^***^ (-0.223 – -0.219) | -0.197 ^***^ (-0.199 – -0.195) | -0.225 ^***^ (-0.227 – -0.224) | -0.203 ^***^ (-0.205 – -0.201) | -0.190 ^***^ (-0.192 – -0.188) | -0.206 ^***^ (-0.208 – -0.204) | -0.199 ^***^ (-0.201 – -0.197) | -0.217 ^***^ (-0.219 – -0.215) |
| Retail and Recreation (14-day Lag) | 0.826 ^***^ (0.815 – 0.837) |  | 0.296 ^***^ (0.292 – 0.300) |  |  |  |  |  |
| Grocery Stores and Pharmacies  (14-day Lag) | -0.354 ^***^ (-0.361 – -0.348) |  |  | 0.092 ^***^ (0.089 – 0.096) |  |  |  |  |
| Parks  (14-day Lag) | -0.536 ^***^ (-0.543 – -0.530) |  |  |  | -0.039 ^***^ (-0.043 – -0.034) |  |  |  |
| Transit Stations  (14-day Lag) | -0.134 ^***^ (-0.143 – -0.125) |  |  |  |  | 0.183 ^***^ (0.178 – 0.188) |  |  |
| Workplaces  (14-day Lag) | -0.592 ^***^ (-0.599 – -0.585) |  |  |  |  |  | 0.089 ^***^ (0.087 – 0.092) |  |
| Residential  (14-day Lag) | -0.601 ^***^ (-0.611 – -0.591) |  |  |  |  |  |  | -0.185 ^***^ (-0.187 – -0.182) |
| Observations | 9557 | 9557 | 9557 | 9557 | 9557 | 9557 | 9557 | 9557 |
| ** p<0.05   ** p<0.01   *** p<0.001* | | | | | | | | |

| **Table S2.** GLM Regression against new cases for Low 2 cluster for the entire year. The 95% confidence intervals are shown in parenthesis. Estimated coefficients for county-level fixed effects and epidemiological terms (immunity factor and lagged daily cases) are not shown. | | | | | | | | |
| --- | --- | --- | --- | --- | --- | --- | --- | --- |
|  | **All**  **[Eq 1]** | **Absolute Humidity**  **[Eq 2]** | **Retail and Rec**  **[Eq 3]** | **Grocery and Pharmacy**  **[Eq 4]** | **Parks**  **[Eq 5]** | **Transit**  **[Eq 6]** | **Workplaces**  **[Eq 7]** | **Residential**  **[Eq 8]** |
| *Predictors* | *Log-Mean* | *Log-Mean* | *Log-Mean* | *Log-Mean* | *Log-Mean* | *Log-Mean* | *Log-Mean* | *Log-Mean* |
| Intercept | 3.439 ^***^ (3.423 – 3.455) | 3.111 ^***^ (3.100 – 3.123) | 3.365 ^***^ (3.353 – 3.377) | 3.203 ^***^ (3.191 – 3.215) | 3.191 ^***^ (3.176 – 3.205) | 3.114 ^***^ (3.102 – 3.125) | 3.160 ^***^ (3.148 – 3.171) | 3.228 ^***^ (3.217 – 3.240) |
| Absolute Humidity  (14-day Lag) | -0.084 ^***^ (-0.085 – -0.082) | -0.030 ^***^ (-0.031 – -0.029) | -0.050 ^***^ (-0.051 – -0.049) | -0.036 ^***^ (-0.037 – -0.035) | -0.039 ^***^ (-0.041 – -0.038) | -0.031 ^***^ (-0.032 – -0.031) | -0.031 ^***^ (-0.031 – -0.030) | -0.042 ^***^ (-0.043 – -0.041) |
| Retail and Recreation (14-day Lag) | 0.839 ^***^ (0.829 – 0.850) |  | 0.245 ^***^ (0.242 – 0.249) |  |  |  |  |  |
| Grocery Stores and Pharmacies  (14-day Lag) | -0.145 ^***^ (-0.152 – -0.138) |  |  | 0.119 ^***^ (0.115 – 0.123) |  |  |  |  |
| Parks  (14-day Lag) | -0.123 ^***^ (-0.128 – -0.118) |  |  |  | 0.042 ^***^ (0.037 – 0.046) |  |  |  |
| Transit Stations  (14-day Lag) | -0.519 ^***^ (-0.528 – -0.511) |  |  |  |  | 0.035 ^***^ (0.030 – 0.040) |  |  |
| Workplaces  (14-day Lag) | -0.560 ^***^ (-0.569 – -0.552) |  |  |  |  |  | 0.041 ^***^ (0.039 – 0.044) |  |
| Residential  (14-day Lag) | -0.425 ^***^ (-0.437 – -0.413) |  |  |  |  |  |  | -0.117 ^***^ (-0.120 – -0.115) |
| Observations | 7987 | 7987 | 7987 | 7987 | 7987 | 7987 | 7987 | 7987 |
| ** p<0.05   ** p<0.01   *** p<0.001* | | | | | | | | |

| **Table S3.** GLM Regression against new cases for Mid 1 cluster for the entire year. The 95% confidence intervals are shown in parenthesis. Estimated coefficients for county-level fixed effects and epidemiological terms (immunity factor and lagged daily cases) are not shown. | | | | | | | | |
| --- | --- | --- | --- | --- | --- | --- | --- | --- |
|  | **All**  **[Eq 1]** | **Absolute Humidity**  **[Eq 2]** | **Retail and Rec**  **[Eq 3]** | **Grocery and Pharmacy**  **[Eq 4]** | **Parks**  **[Eq 5]** | **Transit**  **[Eq 6]** | **Workplaces**  **[Eq 7]** | **Residential**  **[Eq 8]** |
| *Predictors* | *Log-Mean* | *Log-Mean* | *Log-Mean* | *Log-Mean* | *Log-Mean* | *Log-Mean* | *Log-Mean* | *Log-Mean* |
| Intercept | 3.735 ^***^ (3.730 – 3.740) | 3.545 ^***^ (3.541 – 3.550) | 3.762 ^***^ (3.757 – 3.766) | 3.688 ^***^ (3.684 – 3.693) | 3.516 ^***^ (3.511 – 3.521) | 3.498 ^***^ (3.493 – 3.502) | 3.546 ^***^ (3.542 – 3.551) | 3.620 ^***^ (3.615 – 3.624) |
| Absolute Humidity  (14-day Lag) | -0.123 ^***^ (-0.124 – -0.123) | -0.091 ^***^ (-0.091 – -0.090) | -0.101 ^***^ (-0.101 – -0.100) | -0.095 ^***^ (-0.095 – -0.094) | -0.087 ^***^ (-0.088 – -0.087) | -0.089 ^***^ (-0.089 – -0.089) | -0.091 ^***^ (-0.091 – -0.090) | -0.095 ^***^ (-0.095 – -0.094) |
| Retail and Recreation (14-day Lag) | 0.925 ^***^ (0.920 – 0.930) |  | 0.176 ^***^ (0.175 – 0.177) |  |  |  |  |  |
| Grocery Stores and Pharmacies  (14-day Lag) | 0.040 ^***^ (0.037 – 0.042) |  |  | 0.187 ^***^ (0.185 – 0.188) |  |  |  |  |
| Parks  (14-day Lag) | -0.156 ^***^ (-0.159 – -0.154) |  |  |  | -0.018 ^***^ (-0.020 – -0.017) |  |  |  |
| Transit Stations  (14-day Lag) | -0.762 ^***^ (-0.766 – -0.758) |  |  |  |  | -0.056 ^***^ (-0.058 – -0.054) |  |  |
| Workplaces  (14-day Lag) | -0.386 ^***^ (-0.390 – -0.383) |  |  |  |  |  | 0.001 ^*^ (0.000 – 0.002) |  |
| Residential  (14-day Lag) | -0.166 ^***^ (-0.171 – -0.161) |  |  |  |  |  |  | -0.055 ^***^ (-0.056 – -0.054) |
| Observations | 25568 | 25568 | 25568 | 25568 | 25568 | 25568 | 25568 | 25568 |
| ** p<0.05   ** p<0.01   *** p<0.001* | | | | | | | | |

| **Table S4.** GLM Regression against new cases for Mid 2 cluster for the entire year. The 95% confidence intervals are shown in parenthesis. Estimated coefficients for county-level fixed effects and epidemiological terms (immunity factor and lagged daily cases) are not shown. | | | | | | | | |
| --- | --- | --- | --- | --- | --- | --- | --- | --- |
|  | **All**  **[Eq 1]** | **Absolute Humidity**  **[Eq 2]** | **Retail and Rec**  **[Eq 3]** | **Grocery and Pharmacy**  **[Eq 4]** | **Parks**  **[Eq 5]** | **Transit**  **[Eq 6]** | **Workplaces**  **[Eq 7]** | **Residential**  **[Eq 8]** |
| *Predictors* | *Log-Mean* | *Log-Mean* | *Log-Mean* | *Log-Mean* | *Log-Mean* | *Log-Mean* | *Log-Mean* | *Log-Mean* |
| Intercept | 3.885 ^***^ (3.876 – 3.894) | 3.585 ^***^ (3.577 – 3.592) | 3.813 ^***^ (3.805 – 3.821) | 3.781 ^***^ (3.774 – 3.789) | 3.554 ^***^ (3.546 – 3.562) | 3.456 ^***^ (3.448 – 3.465) | 3.558 ^***^ (3.550 – 3.565) | 3.691 ^***^ (3.683 – 3.699) |
| Absolute Humidity  (14-day Lag) | -0.171 ^***^ (-0.171 – -0.170) | -0.134 ^***^ (-0.134 – -0.133) | -0.135 ^***^ (-0.135 – -0.135) | -0.135 ^***^ (-0.135 – -0.134) | -0.131 ^***^ (-0.131 – -0.130) | -0.133 ^***^ (-0.134 – -0.133) | -0.134 ^***^ (-0.135 – -0.134) | -0.135 ^***^ (-0.135 – -0.135) |
| Retail and Recreation (14-day Lag) | 0.515 ^***^ (0.511 – 0.519) |  | 0.119 ^***^ (0.118 – 0.120) |  |  |  |  |  |
| Grocery Stores and Pharmacies  (14-day Lag) | 0.171 ^***^ (0.169 – 0.174) |  |  | 0.152 ^***^ (0.151 – 0.154) |  |  |  |  |
| Parks  (14-day Lag) | -0.200 ^***^ (-0.202 – -0.198) |  |  |  | -0.020 ^***^ (-0.022 – -0.018) |  |  |  |
| Transit Stations  (14-day Lag) | -0.602 ^***^ (-0.607 – -0.598) |  |  |  |  | -0.070 ^***^ (-0.072 – -0.069) |  |  |
| Workplaces  (14-day Lag) | -0.683 ^***^ (-0.686 – -0.680) |  |  |  |  |  | -0.017 ^***^ (-0.018 – -0.016) |  |
| Residential  (14-day Lag) | -0.576 ^***^ (-0.580 – -0.572) |  |  |  |  |  |  | -0.043 ^***^ (-0.044 – -0.042) |
| Observations | 27087 | 27087 | 27087 | 27087 | 27087 | 27087 | 27087 | 27087 |
| ** p<0.05   ** p<0.01   *** p<0.001* | | | | | | | | |

| **Table S5.** GLM Regression against new cases for High 1 cluster for the entire year. The 95% confidence intervals are shown in parenthesis. Estimated coefficients for county-level fixed effects and epidemiological terms (immunity factor and lagged daily cases) are not shown. | | | | | | | | |
| --- | --- | --- | --- | --- | --- | --- | --- | --- |
|  | **All**  **[Eq 1]** | **Absolute Humidity**  **[Eq 2]** | **Retail and Rec**  **[Eq 3]** | **Grocery and Pharmacy**  **[Eq 4]** | **Parks**  **[Eq 5]** | **Transit**  **[Eq 6]** | **Workplaces**  **[Eq 7]** | **Residential**  **[Eq 8]** |
| *Predictors* | *Log-Mean* | *Log-Mean* | *Log-Mean* | *Log-Mean* | *Log-Mean* | *Log-Mean* | *Log-Mean* | *Log-Mean* |
| Intercept | 4.381 ^***^ (4.291 – 4.469) | 2.762 ^***^ (2.672 – 2.849) | 3.019 ^***^ (2.929 – 3.106) | 2.777 ^***^ (2.688 – 2.865) | 2.726 ^***^ (2.637 – 2.814) | 2.772 ^***^ (2.682 – 2.859) | 2.741 ^***^ (2.652 – 2.829) | 2.803 ^***^ (2.713 – 2.890) |
| Absolute Humidity  (14-day Lag) | -0.060 ^***^ (-0.060 – -0.059) | -0.027 ^***^ (-0.028 – -0.027) | -0.038 ^***^ (-0.038 – -0.037) | -0.030 ^***^ (-0.030 – -0.029) | -0.015 ^***^ (-0.016 – -0.015) | -0.026 ^***^ (-0.026 – -0.025) | -0.025 ^***^ (-0.026 – -0.025) | -0.030 ^***^ (-0.031 – -0.030) |
| Retail and Recreation (14-day Lag) | 0.950 ^***^ (0.941 – 0.959) |  | 0.212 ^***^ (0.209 – 0.214) |  |  |  |  |  |
| Grocery Stores and Pharmacies  (14-day Lag) | -0.223 ^***^ (-0.228 – -0.217) |  |  | 0.123 ^***^ (0.120 – 0.126) |  |  |  |  |
| Parks  (14-day Lag) | -0.379 ^***^ (-0.383 – -0.374) |  |  |  | -0.121 ^***^ (-0.124 – -0.118) |  |  |  |
| Transit Stations  (14-day Lag) | -0.350 ^***^ (-0.356 – -0.344) |  |  |  |  | -0.056 ^***^ (-0.060 – -0.052) |  |  |
| Workplaces  (14-day Lag) | -0.762 ^***^ (-0.767 – -0.757) |  |  |  |  |  | -0.081 ^***^ (-0.083 – -0.079) |  |
| Residential  (14-day Lag) | -0.583 ^***^ (-0.591 – -0.575) |  |  |  |  |  |  | -0.048 ^***^ (-0.050 – -0.047) |
| Observations | 16581 | 16581 | 16581 | 16581 | 16581 | 16581 | 16581 | 16581 |
| ** p<0.05   ** p<0.01   *** p<0.001* | | | | | | | | |

| **Table S6.** GLM Regression against new cases for High 2 cluster for the entire year. The 95% confidence intervals are shown in parenthesis. Estimated coefficients for county-level fixed effects and epidemiological terms (immunity factor and lagged daily cases) are not shown. | | | | | | | | |
| --- | --- | --- | --- | --- | --- | --- | --- | --- |
|  | **All**  **[Eq 1]** | **Absolute Humidity**  **[Eq 2]** | **Retail and Rec**  **[Eq 3]** | **Grocery and Pharmacy**  **[Eq 4]** | **Parks**  **[Eq 5]** | **Transit**  **[Eq 6]** | **Workplaces**  **[Eq 7]** | **Residential**  **[Eq 8]** |
| *Predictors* | *Log-Mean* | *Log-Mean* | *Log-Mean* | *Log-Mean* | *Log-Mean* | *Log-Mean* | *Log-Mean* | *Log-Mean* |
| Intercept | 3.270 ^***^ (3.254 – 3.285) | 2.950 ^***^ (2.935 – 2.965) | 3.021 ^***^ (3.006 – 3.036) | 2.876 ^***^ (2.861 – 2.891) | 2.874 ^***^ (2.859 – 2.889) | 2.948 ^***^ (2.933 – 2.963) | 2.943 ^***^ (2.928 – 2.958) | 2.967 ^***^ (2.952 – 2.982) |
| Absolute Humidity  (14-day Lag) | -0.015 ^***^ (-0.015 – -0.015) | -0.010 ^***^ (-0.011 – -0.010) | -0.014 ^***^ (-0.014 – -0.014) | -0.010 ^***^ (-0.010 – -0.009) | -0.004 ^***^ (-0.004 – -0.003) | -0.011 ^***^ (-0.011 – -0.010) | -0.010 ^***^ (-0.011 – -0.010) | -0.012 ^***^ (-0.013 – -0.012) |
| Retail and Recreation (14-day Lag) | 1.299 ^***^ (1.293 – 1.305) |  | 0.204 ^***^ (0.202 – 0.206) |  |  |  |  |  |
| Grocery Stores and Pharmacies  (14-day Lag) | -0.130 ^***^ (-0.134 – -0.126) |  |  | 0.119 ^***^ (0.117 – 0.121) |  |  |  |  |
| Parks  (14-day Lag) | -0.984 ^***^ (-0.988 – -0.979) |  |  |  | -0.186 ^***^ (-0.189 – -0.183) |  |  |  |
| Transit Stations  (14-day Lag) | -0.339 ^***^ (-0.343 – -0.335) |  |  |  |  | 0.032 ^***^ (0.029 – 0.034) |  |  |
| Workplaces  (14-day Lag) | -0.541 ^***^ (-0.544 – -0.538) |  |  |  |  |  | -0.028 ^***^ (-0.029 – -0.026) |  |
| Residential  (14-day Lag) | -0.269 ^***^ (-0.273 – -0.265) |  |  |  |  |  |  | -0.070 ^***^ (-0.071 – -0.068) |
| Observations | 25916 | 25916 | 25916 | 25916 | 25916 | 25916 | 25916 | 25916 |
| ** p<0.05   ** p<0.01   *** p<0.001* | | | | | | | | |

| **Table S7.** GLM Regression against new cases for Low 1 cluster from March 10, 2020 to September 30, 2020. The 95% confidence intervals are shown in parenthesis. Estimated coefficients for county-level fixed effects and epidemiological terms (immunity factor and lagged daily cases) are not shown. | | | | | | | | |
| --- | --- | --- | --- | --- | --- | --- | --- | --- |
|  | **All**  **[Eq 1]** | **Absolute Humidity**  **[Eq 2]** | **Retail and Rec**  **[Eq 3]** | **Grocery and Pharmacy**  **[Eq 4]** | **Parks**  **[Eq 5]** | **Transit**  **[Eq 6]** | **Workplaces**  **[Eq 7]** | **Residential**  **[Eq 8]** |
| *Predictors* | *Log-Mean* | *Log-Mean* | *Log-Mean* | *Log-Mean* | *Log-Mean* | *Log-Mean* | *Log-Mean* | *Log-Mean* |
| Intercept | 2.064 ^***^ (1.982 – 2.145) | 1.367 ^***^ (1.285 – 1.446) | 1.523 ^***^ (1.441 – 1.603) | 1.387 ^***^ (1.306 – 1.467) | 1.339 ^***^ (1.257 – 1.418) | 1.424 ^***^ (1.342 – 1.504) | 1.379 ^***^ (1.298 – 1.459) | 1.481 ^***^ (1.399 – 1.561) |
| Absolute Humidity  (14-day Lag) | -0.069 ^***^ (-0.073 – -0.065) | -0.032 ^***^ (-0.035 – -0.029) | -0.046 ^***^ (-0.049 – -0.043) | -0.037 ^***^ (-0.040 – -0.033) | -0.050 ^***^ (-0.053 – -0.047) | -0.035 ^***^ (-0.038 – -0.032) | -0.033 ^***^ (-0.036 – -0.029) | -0.042 ^***^ (-0.046 – -0.039) |
| Retail and Recreation (14-day Lag) | 1.313 ^***^ (1.279 – 1.348) |  | 0.153 ^***^ (0.146 – 0.161) |  |  |  |  |  |
| Grocery Stores and Pharmacies  (14-day Lag) | -0.148 ^***^ (-0.166 – -0.130) |  |  | 0.078 ^***^ (0.070 – 0.085) |  |  |  |  |
| Parks  (14-day Lag) | -0.545 ^***^ (-0.567 – -0.523) |  |  |  | 0.161 ^***^ (0.148 – 0.173) |  |  |  |
| Transit Stations  (14-day Lag) | -0.463 ^***^ (-0.497 – -0.430) |  |  |  |  | 0.084 ^***^ (0.073 – 0.095) |  |  |
| Workplaces  (14-day Lag) | -0.650 ^***^ (-0.669 – -0.631) |  |  |  |  |  | 0.011 ^***^ (0.005 – 0.016) |  |
| Residential  (14-day Lag) | -0.256 ^***^ (-0.278 – -0.233) |  |  |  |  |  |  | -0.077 ^***^ (-0.082 – -0.072) |
| Observations | 3604 | 3604 | 3604 | 3604 | 3604 | 3604 | 3604 | 3604 |
| ** p<0.05   ** p<0.01   *** p<0.001* | | | | | | | | |

| **Table S8.** GLM Regression against new cases for Low 2 cluster from March 10, 2020 to September 30, 2020. The 95% confidence intervals are shown in parenthesis. Estimated coefficients for county-level fixed effects and epidemiological terms (immunity factor and lagged daily cases) are not shown. | | | | | | | | |
| --- | --- | --- | --- | --- | --- | --- | --- | --- |
|  | **All**  **[Eq 1]** | **Absolute Humidity**  **[Eq 2]** | **Retail and Rec**  **[Eq 3]** | **Grocery and Pharmacy**  **[Eq 4]** | **Parks**  **[Eq 5]** | **Transit**  **[Eq 6]** | **Workplaces**  **[Eq 7]** | **Residential**  **[Eq 8]** |
| *Predictors* | *Log-Mean* | *Log-Mean* | *Log-Mean* | *Log-Mean* | *Log-Mean* | *Log-Mean* | *Log-Mean* | *Log-Mean* |
| Intercept | 2.198 ^***^ (2.159 – 2.236) | 1.968 ^***^ (1.943 – 1.994) | 1.671 ^***^ (1.642 – 1.700) | 1.767 ^***^ (1.739 – 1.794) | 2.384 ^***^ (2.346 – 2.422) | 1.650 ^***^ (1.623 – 1.677) | 1.722 ^***^ (1.695 – 1.749) | 1.726 ^***^ (1.698 – 1.753) |
| Absolute Humidity  (14-day Lag) | -0.038 ^***^ (-0.041 – -0.035) | -0.004 ^***^ (-0.007 – -0.002) | 0.016 ^***^ (0.014 – 0.018) | 0.009 ^***^ (0.007 – 0.011) | -0.029 ^***^ (-0.032 – -0.027) | 0.020 ^***^ (0.018 – 0.022) | 0.010 ^***^ (0.007 – 0.012) | 0.017 ^***^ (0.015 – 0.019) |
| Retail and Recreation (14-day Lag) | 0.276 ^***^ (0.247 – 0.305) |  | -0.203 ^***^ (-0.212 – -0.195) |  |  |  |  |  |
| Grocery Stores and Pharmacies  (14-day Lag) | 0.096 ^***^ (0.079 – 0.113) |  |  | -0.170 ^***^ (-0.179 – -0.161) |  |  |  |  |
| Parks  (14-day Lag) | 0.098 ^***^ (0.085 – 0.111) |  |  |  | 0.159 ^***^ (0.148 – 0.170) |  |  |  |
| Transit Stations  (14-day Lag) | -1.021 ^***^ (-1.052 – -0.989) |  |  |  |  | -0.500 ^***^ (-0.514 – -0.486) |  |  |
| Workplaces  (14-day Lag) | -0.544 ^***^ (-0.574 – -0.514) |  |  |  |  |  | -0.192 ^***^ (-0.198 – -0.185) |  |
| Residential  (14-day Lag) | -0.579 ^***^ (-0.615 – -0.543) |  |  |  |  |  |  | 0.174 ^***^ (0.167 – 0.180) |
| Observations | 2903 | 2903 | 2903 | 2903 | 2903 | 2903 | 2903 | 2903 |
| ** p<0.05   ** p<0.01   *** p<0.001* | | | | | | | | |

| **Table S9.** GLM Regression against new cases for Mid 1 cluster from March 10, 2020 to September 30, 2020. The 95% confidence intervals are shown in parenthesis. Estimated coefficients for county-level fixed effects and epidemiological terms (immunity factor and lagged daily cases) are not shown. | | | | | | | | |
| --- | --- | --- | --- | --- | --- | --- | --- | --- |
|  | **All**  **[Eq 1]** | **Absolute Humidity**  **[Eq 2]** | **Retail and Rec**  **[Eq 3]** | **Grocery and Pharmacy**  **[Eq 4]** | **Parks**  **[Eq 5]** | **Transit**  **[Eq 6]** | **Workplaces**  **[Eq 7]** | **Residential**  **[Eq 8]** |
| *Predictors* | *Log-Mean* | *Log-Mean* | *Log-Mean* | *Log-Mean* | *Log-Mean* | *Log-Mean* | *Log-Mean* | *Log-Mean* |
| Intercept | 3.064 ^***^ (3.053 – 3.074) | 2.872 ^***^ (2.865 – 2.878) | 2.939 ^***^ (2.932 – 2.946) | 2.954 ^***^ (2.947 – 2.961) | 2.988 ^***^ (2.979 – 2.998) | 2.857 ^***^ (2.850 – 2.865) | 2.866 ^***^ (2.859 – 2.873) | 2.889 ^***^ (2.882 – 2.896) |
| Absolute Humidity  (14-day Lag) | -0.098 ^***^ (-0.099 – -0.097) | -0.050 ^***^ (-0.051 – -0.049) | -0.056 ^***^ (-0.057 – -0.056) | -0.057 ^***^ (-0.058 – -0.057) | -0.061 ^***^ (-0.062 – -0.060) | -0.050 ^***^ (-0.050 – -0.049) | -0.050 ^***^ (-0.050 – -0.049) | -0.052 ^***^ (-0.053 – -0.051) |
| Retail and Recreation (14-day Lag) | 0.709 ^***^ (0.700 – 0.719) |  | 0.059 ^***^ (0.057 – 0.061) |  |  |  |  |  |
| Grocery Stores and Pharmacies  (14-day Lag) | 0.352 ^***^ (0.347 – 0.357) |  |  | 0.125 ^***^ (0.123 – 0.128) |  |  |  |  |
| Parks  (14-day Lag) | -0.184 ^***^ (-0.190 – -0.179) |  |  |  | 0.068 ^***^ (0.064 – 0.072) |  |  |  |
| Transit Stations  (14-day Lag) | -0.633 ^***^ (-0.645 – -0.621) |  |  |  |  | -0.018 ^***^ (-0.022 – -0.015) |  |  |
| Workplaces  (14-day Lag) | -0.696 ^***^ (-0.705 – -0.687) |  |  |  |  |  | -0.007 ^***^ (-0.009 – -0.006) |  |
| Residential  (14-day Lag) | -0.356 ^***^ (-0.367 – -0.345) |  |  |  |  |  |  | -0.015 ^***^ (-0.016 – -0.013) |
| Observations | 9781 | 9781 | 9781 | 9781 | 9781 | 9781 | 9781 | 9781 |
| ** p<0.05   ** p<0.01   *** p<0.001* | | | | | | | | |

| **Table S10.** GLM Regression against new cases for Mid 2 cluster from March 10, 2020 to September 30, 2020. The 95% confidence intervals are shown in parenthesis. Estimated coefficients for county-level fixed effects and epidemiological terms (immunity factor and lagged daily cases) are not shown. | | | | | | | | |
| --- | --- | --- | --- | --- | --- | --- | --- | --- |
|  | **All**  **[Eq 1]** | **Absolute Humidity**  **[Eq 2]** | **Retail and Rec**  **[Eq 3]** | **Grocery and Pharmacy**  **[Eq 4]** | **Parks**  **[Eq 5]** | **Transit**  **[Eq 6]** | **Workplaces**  **[Eq 7]** | **Residential**  **[Eq 8]** |
| *Predictors* | *Log-Mean* | *Log-Mean* | *Log-Mean* | *Log-Mean* | *Log-Mean* | *Log-Mean* | *Log-Mean* | *Log-Mean* |
| Intercept | 3.033 ^***^ (3.014 – 3.053) | 2.379 ^***^ (2.364 – 2.393) | 2.577 ^***^ (2.562 – 2.593) | 2.660 ^***^ (2.644 – 2.675) | 2.414 ^***^ (2.399 – 2.430) | 2.476 ^***^ (2.461 – 2.492) | 2.439 ^***^ (2.424 – 2.454) | 2.525 ^***^ (2.510 – 2.541) |
| Absolute Humidity  (14-day Lag) | -0.108 ^***^ (-0.109 – -0.106) | -0.053 ^***^ (-0.054 – -0.052) | -0.060 ^***^ (-0.061 – -0.059) | -0.065 ^***^ (-0.066 – -0.064) | -0.056 ^***^ (-0.057 – -0.055) | -0.055 ^***^ (-0.055 – -0.054) | -0.055 ^***^ (-0.055 – -0.054) | -0.059 ^***^ (-0.060 – -0.058) |
| Retail and Recreation (14-day Lag) | 0.288 ^***^ (0.280 – 0.296) |  | 0.074 ^***^ (0.073 – 0.076) |  |  |  |  |  |
| Grocery Stores and Pharmacies  (14-day Lag) | 0.492 ^***^ (0.487 – 0.496) |  |  | 0.177 ^***^ (0.175 – 0.179) |  |  |  |  |
| Parks  (14-day Lag) | -0.239 ^***^ (-0.244 – -0.235) |  |  |  | 0.025 ^***^ (0.021 – 0.029) |  |  |  |
| Transit Stations  (14-day Lag) | -0.589 ^***^ (-0.603 – -0.576) |  |  |  |  | 0.051 ^***^ (0.048 – 0.054) |  |  |
| Workplaces  (14-day Lag) | -0.900 ^***^ (-0.909 – -0.892) |  |  |  |  |  | 0.026 ^***^ (0.024 – 0.027) |  |
| Residential  (14-day Lag) | -0.782 ^***^ (-0.791 – -0.773) |  |  |  |  |  |  | -0.043 ^***^ (-0.045 – -0.042) |
| Observations | 11260 | 11260 | 11260 | 11260 | 11260 | 11260 | 11260 | 11260 |
| ** p<0.05   ** p<0.01   *** p<0.001* | | | | | | | | |

| **Table S11.** GLM Regression against new cases for High 1 cluster from March 10, 2020 to September 30, 2020. The 95% confidence intervals are shown in parenthesis. Estimated coefficients for county-level fixed effects and epidemiological terms (immunity factor and lagged daily cases) are not shown. | | | | | | | | |
| --- | --- | --- | --- | --- | --- | --- | --- | --- |
|  | **All**  **[Eq 1]** | **Absolute Humidity**  **[Eq 2]** | **Retail and Rec**  **[Eq 3]** | **Grocery and Pharmacy**  **[Eq 4]** | **Parks**  **[Eq 5]** | **Transit**  **[Eq 6]** | **Workplaces**  **[Eq 7]** | **Residential**  **[Eq 8]** |
| *Predictors* | *Log-Mean* | *Log-Mean* | *Log-Mean* | *Log-Mean* | *Log-Mean* | *Log-Mean* | *Log-Mean* | *Log-Mean* |
| Intercept | 1.020 ^***^ (0.952 – 1.087) | 0.007  (-0.058 – 0.071) | 0.064  (-0.003 – 0.128) | -0.035  (-0.101 – 0.029) | -0.072 ^*^ (-0.138 – -0.008) | -0.101 ^**^ (-0.167 – -0.037) | -0.081 ^*^ (-0.146 – -0.016) | -0.082 ^*^ (-0.148 – -0.018) |
| Absolute Humidity  (14-day Lag) | 0.071 ^***^ (0.069 – 0.073) | 0.106 ^***^ (0.104 – 0.107) | 0.103 ^***^ (0.102 – 0.105) | 0.111 ^***^ (0.110 – 0.113) | 0.113 ^***^ (0.111 – 0.114) | 0.110 ^***^ (0.108 – 0.111) | 0.110 ^***^ (0.109 – 0.112) | 0.110 ^***^ (0.109 – 0.112) |
| Retail and Recreation (14-day Lag) | 0.866 ^***^ (0.847 – 0.884) |  | 0.032 ^***^ (0.026 – 0.038) |  |  |  |  |  |
| Grocery Stores and Pharmacies  (14-day Lag) | -0.112 ^***^ (-0.125 – -0.098) |  |  | -0.090 ^***^ (-0.098 – -0.083) |  |  |  |  |
| Parks  (14-day Lag) | -0.144 ^***^ (-0.153 – -0.136) |  |  |  | -0.066 ^***^ (-0.073 – -0.059) |  |  |  |
| Transit Stations  (14-day Lag) | -0.230 ^***^ (-0.247 – -0.213) |  |  |  |  | -0.141 ^***^ (-0.150 – -0.132) |  |  |
| Workplaces  (14-day Lag) | -0.644 ^***^ (-0.663 – -0.625) |  |  |  |  |  | -0.089 ^***^ (-0.093 – -0.085) |  |
| Residential  (14-day Lag) | -0.233 ^***^ (-0.256 – -0.209) |  |  |  |  |  |  | 0.055 ^***^ (0.050 – 0.059) |
| Observations | 6446 | 6446 | 6446 | 6446 | 6446 | 6446 | 6446 | 6446 |
| ** p<0.05   ** p<0.01   *** p<0.001* | | | | | | | | |

| **Table S12.** GLM Regression against new cases for High 2 cluster from March 10, 2020 to September 30, 2020. The 95% confidence intervals are shown in parenthesis. Estimated coefficients for county-level fixed effects and epidemiological terms (immunity factor and lagged daily cases) are not shown. | | | | | | | | |
| --- | --- | --- | --- | --- | --- | --- | --- | --- |
|  | **All**  **[Eq 1]** | **Absolute Humidity**  **[Eq 2]** | **Retail and Rec**  **[Eq 3]** | **Grocery and Pharmacy**  **[Eq 4]** | **Parks**  **[Eq 5]** | **Transit**  **[Eq 6]** | **Workplaces**  **[Eq 7]** | **Residential**  **[Eq 8]** |
| *Predictors* | *Log-Mean* | *Log-Mean* | *Log-Mean* | *Log-Mean* | *Log-Mean* | *Log-Mean* | *Log-Mean* | *Log-Mean* |
| Intercept | -2.835 ^***^ (-2.875 – -2.795) | -1.807 ^***^ (-1.841 – -1.773) | -2.043 ^***^ (-2.078 – -2.009) | -2.775 ^***^ (-2.810 – -2.739) | -2.857 ^***^ (-2.893 – -2.822) | -2.193 ^***^ (-2.228 – -2.159) | -1.945 ^***^ (-1.979 – -1.912) | -1.882 ^***^ (-1.916 – -1.849) |
| Absolute Humidity  (14-day Lag) | 0.221 ^***^ (0.220 – 0.222) | 0.235 ^***^ (0.234 – 0.236) | 0.229 ^***^ (0.228 – 0.230) | 0.246 ^***^ (0.245 – 0.247) | 0.232 ^***^ (0.231 – 0.232) | 0.240 ^***^ (0.239 – 0.241) | 0.242 ^***^ (0.241 – 0.242) | 0.236 ^***^ (0.235 – 0.236) |
| Retail and Recreation (14-day Lag) | 0.537 ^***^ (0.523 – 0.550) |  | 0.404 ^***^ (0.401 – 0.408) |  |  |  |  |  |
| Grocery Stores and Pharmacies  (14-day Lag) | 0.261 ^***^ (0.253 – 0.269) |  |  | 0.404 ^***^ (0.401 – 0.408) |  |  |  |  |
| Parks  (14-day Lag) | 0.528 ^***^ (0.514 – 0.541) |  |  |  | 0.849 ^***^ (0.842 – 0.857) |  |  |  |
| Transit Stations  (14-day Lag) | -0.411 ^***^ (-0.423 – -0.400) |  |  |  |  | 0.385 ^***^ (0.380 – 0.391) |  |  |
| Workplaces  (14-day Lag) | -0.072 ^***^ (-0.083 – -0.061) |  |  |  |  |  | 0.202 ^***^ (0.200 – 0.205) |  |
| Residential  (14-day Lag) | 0.176 ^***^ (0.165 – 0.188) |  |  |  |  |  |  | -0.215 ^***^ (-0.217 – -0.212) |
| Observations | 11460 | 11460 | 11460 | 11460 | 11460 | 11460 | 11460 | 11460 |
| ** p<0.05   ** p<0.01   *** p<0.001* | | | | | | | | |

| **Table S13.** GLM Regression against new cases for Low 1 cluster from October 1, 2020 to March 1, 2021. The 95% confidence intervals are shown in parenthesis. Estimated coefficients for county-level fixed effects and epidemiological terms (immunity factor and lagged daily cases) are not shown. | | | | | | | | |
| --- | --- | --- | --- | --- | --- | --- | --- | --- |
|  | **All**  **[Eq 1]** | **Absolute Humidity**  **[Eq 2]** | **Retail and Rec**  **[Eq 3]** | **Grocery and Pharmacy**  **[Eq 4]** | **Parks**  **[Eq 5]** | **Transit**  **[Eq 6]** | **Workplaces**  **[Eq 7]** | **Residential**  **[Eq 8]** |
| *Predictors* | *Log-Mean* | *Log-Mean* | *Log-Mean* | *Log-Mean* | *Log-Mean* | *Log-Mean* | *Log-Mean* | *Log-Mean* |
| Intercept | 5.411 ^***^ (5.391 – 5.431) | 5.107 ^***^ (5.088 – 5.126) | 5.046 ^***^ (5.027 – 5.064) | 5.165 ^***^ (5.146 – 5.184) | 5.191 ^***^ (5.172 – 5.209) | 5.007 ^***^ (4.988 – 5.026) | 4.985 ^***^ (4.966 – 5.004) | 4.994 ^***^ (4.975 – 5.013) |
| Absolute Humidity  (14-day Lag) | -0.141 ^***^ (-0.144 – -0.138) | -0.205 ^***^ (-0.208 – -0.202) | -0.198 ^***^ (-0.201 – -0.195) | -0.203 ^***^ (-0.206 – -0.201) | -0.167 ^***^ (-0.170 – -0.164) | -0.200 ^***^ (-0.203 – -0.198) | -0.198 ^***^ (-0.201 – -0.195) | -0.182 ^***^ (-0.184 – -0.179) |
| Retail and Recreation (14-day Lag) | 0.329 ^***^ (0.314 – 0.344) |  | -0.188 ^***^ (-0.195 – -0.180) |  |  |  |  |  |
| Grocery Stores and Pharmacies  (14-day Lag) | 0.312 ^***^ (0.302 – 0.322) |  |  | 0.128 ^***^ (0.121 – 0.135) |  |  |  |  |
| Parks  (14-day Lag) | -0.518 ^***^ (-0.527 – -0.509) |  |  |  | -0.429 ^***^ (-0.436 – -0.422) |  |  |  |
| Transit Stations  (14-day Lag) | 0.244 ^***^ (0.230 – 0.257) |  |  |  |  | -0.253 ^***^ (-0.262 – -0.243) |  |  |
| Workplaces  (14-day Lag) | 0.017 ^***^ (0.007 – 0.027) |  |  |  |  |  | -0.259 ^***^ (-0.264 – -0.254) |  |
| Residential  (14-day Lag) | 0.545 ^***^ (0.529 – 0.561) |  |  |  |  |  |  | 0.406 ^***^ (0.400 – 0.411) |
| Observations | 5953 | 5953 | 5953 | 5953 | 5953 | 5953 | 5953 | 5953 |
| ** p<0.05   ** p<0.01   *** p<0.001* | | | | | | | | |

| **Table S14.** GLM Regression against new cases for Low 2 cluster from October 1, 2020 to March 1, 2021. The 95% confidence intervals are shown in parenthesis. Estimated coefficients for county-level fixed effects and epidemiological terms (immunity factor and lagged daily cases) are not shown. | | | | | | | | |
| --- | --- | --- | --- | --- | --- | --- | --- | --- |
|  | **All**  **[Eq 1]** | **Absolute Humidity**  **[Eq 2]** | **Retail and Rec**  **[Eq 3]** | **Grocery and Pharmacy**  **[Eq 4]** | **Parks**  **[Eq 5]** | **Transit**  **[Eq 6]** | **Workplaces**  **[Eq 7]** | **Residential**  **[Eq 8]** |
| *Predictors* | *Log-Mean* | *Log-Mean* | *Log-Mean* | *Log-Mean* | *Log-Mean* | *Log-Mean* | *Log-Mean* | *Log-Mean* |
| Intercept | 6.039 ^***^ (6.013 – 6.066) | 5.064 ^***^ (5.047 – 5.081) | 5.294 ^***^ (5.275 – 5.312) | 5.552 ^***^ (5.534 – 5.571) | 4.864 ^***^ (4.843 – 4.885) | 5.063 ^***^ (5.046 – 5.080) | 4.903 ^***^ (4.885 – 4.921) | 4.998 ^***^ (4.980 – 5.015) |
| Absolute Humidity  (14-day Lag) | -0.093 ^***^ (-0.096 – -0.091) | -0.120 ^***^ (-0.121 – -0.118) | -0.150 ^***^ (-0.152 – -0.148) | -0.161 ^***^ (-0.162 – -0.159) | -0.091 ^***^ (-0.093 – -0.088) | -0.123 ^***^ (-0.125 – -0.121) | -0.110 ^***^ (-0.111 – -0.108) | -0.084 ^***^ (-0.086 – -0.082) |
| Retail and Recreation (14-day Lag) | 0.780 ^***^ (0.764 – 0.795) |  | 0.266 ^***^ (0.259 – 0.274) |  |  |  |  |  |
| Grocery Stores and Pharmacies  (14-day Lag) | 0.380 ^***^ (0.369 – 0.391) |  |  | 0.555 ^***^ (0.547 – 0.563) |  |  |  |  |
| Parks  (14-day Lag) | -0.030 ^***^ (-0.037 – -0.022) |  |  |  | -0.101 ^***^ (-0.107 – -0.095) |  |  |  |
| Transit Stations  (14-day Lag) | 0.129 ^***^ (0.116 – 0.142) |  |  |  |  | 0.050 ^***^ (0.040 – 0.059) |  |  |
| Workplaces  (14-day Lag) | 0.469 ^***^ (0.458 – 0.480) |  |  |  |  |  | -0.174 ^***^ (-0.178 – -0.169) |  |
| Residential  (14-day Lag) | 1.322 ^***^ (1.304 – 1.340) |  |  |  |  |  |  | 0.292 ^***^ (0.286 – 0.298) |
| Observations | 5084 | 5084 | 5084 | 5084 | 5084 | 5084 | 5084 | 5084 |
| ** p<0.05   ** p<0.01   *** p<0.001* | | | | | | | | |

| **Table S15.** GLM Regression against new cases for Mid 1 cluster from October 1, 2020 to March 1, 2021. The 95% confidence intervals are shown in parenthesis. Estimated coefficients for county-level fixed effects and epidemiological terms (immunity factor and lagged daily cases) are not shown. | | | | | | | | |
| --- | --- | --- | --- | --- | --- | --- | --- | --- |
|  | **All**  **[Eq 1]** | **Absolute Humidity**  **[Eq 2]** | **Retail and Rec**  **[Eq 3]** | **Grocery and Pharmacy**  **[Eq 4]** | **Parks**  **[Eq 5]** | **Transit**  **[Eq 6]** | **Workplaces**  **[Eq 7]** | **Residential**  **[Eq 8]** |
| *Predictors* | *Log-Mean* | *Log-Mean* | *Log-Mean* | *Log-Mean* | *Log-Mean* | *Log-Mean* | *Log-Mean* | *Log-Mean* |
| Intercept | 5.782 ^***^ (5.772 – 5.791) | 4.929 ^***^ (4.923 – 4.936) | 5.226 ^***^ (5.219 – 5.234) | 5.540 ^***^ (5.532 – 5.547) | 4.994 ^***^ (4.986 – 5.002) | 4.739 ^***^ (4.731 – 4.747) | 4.671 ^***^ (4.664 – 4.678) | 4.660 ^***^ (4.653 – 4.667) |
| Absolute Humidity  (14-day Lag) | -0.151 ^***^ (-0.152 – -0.150) | -0.142 ^***^ (-0.143 – -0.141) | -0.170 ^***^ (-0.171 – -0.169) | -0.175 ^***^ (-0.176 – -0.175) | -0.151 ^***^ (-0.152 – -0.150) | -0.128 ^***^ (-0.129 – -0.128) | -0.128 ^***^ (-0.129 – -0.127) | -0.104 ^***^ (-0.105 – -0.103) |
| Retail and Recreation (14-day Lag) | 0.567 ^***^ (0.559 – 0.575) |  | 0.257 ^***^ (0.253 – 0.260) |  |  |  |  |  |
| Grocery Stores and Pharmacies  (14-day Lag) | 0.501 ^***^ (0.497 – 0.506) |  |  | 0.604 ^***^ (0.601 – 0.607) |  |  |  |  |
| Parks  (14-day Lag) | 0.267 ^***^ (0.263 – 0.270) |  |  |  | 0.035 ^***^ (0.033 – 0.038) |  |  |  |
| Transit Stations  (14-day Lag) | -0.171 ^***^ (-0.178 – -0.165) |  |  |  |  | -0.202 ^***^ (-0.207 – -0.198) |  |  |
| Workplaces  (14-day Lag) | 0.514 ^***^ (0.509 – 0.519) |  |  |  |  |  | -0.228 ^***^ (-0.230 – -0.226) |  |
| Residential  (14-day Lag) | 1.273 ^***^ (1.265 – 1.281) |  |  |  |  |  |  | 0.300 ^***^ (0.298 – 0.303) |
| Observations | 15787 | 15787 | 15787 | 15787 | 15787 | 15787 | 15787 | 15787 |
| ** p<0.05   ** p<0.01   *** p<0.001* | | | | | | | | |

| **Table S16.** GLM Regression against new cases for Mid 2 cluster from October 1, 2020 to March 1, 2021. The 95% confidence intervals are shown in parenthesis. Estimated coefficients for county-level fixed effects and epidemiological terms (immunity factor and lagged daily cases) are not shown. | | | | | | | | |
| --- | --- | --- | --- | --- | --- | --- | --- | --- |
|  | **All**  **[Eq 1]** | **Absolute Humidity**  **[Eq 2]** | **Retail and Rec**  **[Eq 3]** | **Grocery and Pharmacy**  **[Eq 4]** | **Parks**  **[Eq 5]** | **Transit**  **[Eq 6]** | **Workplaces**  **[Eq 7]** | **Residential**  **[Eq 8]** |
| *Predictors* | *Log-Mean* | *Log-Mean* | *Log-Mean* | *Log-Mean* | *Log-Mean* | *Log-Mean* | *Log-Mean* | *Log-Mean* |
| Intercept | 4.553 ^***^ (4.540 – 4.566) | 4.924 ^***^ (4.915 – 4.933) | 4.938 ^***^ (4.926 – 4.950) | 5.638 ^***^ (5.628 – 5.649) | 4.961 ^***^ (4.951 – 4.971) | 4.594 ^***^ (4.581 – 4.606) | 4.369 ^***^ (4.358 – 4.379) | 4.086 ^***^ (4.075 – 4.097) |
| Absolute Humidity  (14-day Lag) | -0.220 ^***^ (-0.221 – -0.219) | -0.225 ^***^ (-0.225 – -0.224) | -0.225 ^***^ (-0.226 – -0.224) | -0.239 ^***^ (-0.240 – -0.239) | -0.228 ^***^ (-0.229 – -0.228) | -0.219 ^***^ (-0.219 – -0.218) | -0.219 ^***^ (-0.219 – -0.218) | -0.199 ^***^ (-0.199 – -0.198) |
| Retail and Recreation (14-day Lag) | -0.167 ^***^ (-0.175 – -0.158) |  | 0.007 ^***^ (0.003 – 0.011) |  |  |  |  |  |
| Grocery Stores and Pharmacies  (14-day Lag) | 0.782 ^***^ (0.777 – 0.788) |  |  | 0.461 ^***^ (0.458 – 0.464) |  |  |  |  |
| Parks  (14-day Lag) | 0.277 ^***^ (0.274 – 0.281) |  |  |  | 0.024 ^***^ (0.021 – 0.026) |  |  |  |
| Transit Stations  (14-day Lag) | -0.023 ^***^ (-0.027 – -0.019) |  |  |  |  | -0.155 ^***^ (-0.159 – -0.151) |  |  |
| Workplaces  (14-day Lag) | 0.347 ^***^ (0.342 – 0.352) |  |  |  |  |  | -0.294 ^***^ (-0.296 – -0.292) |  |
| Residential  (14-day Lag) | 0.931 ^***^ (0.923 – 0.938) |  |  |  |  |  |  | 0.343 ^***^ (0.340 – 0.345) |
| Observations | 15827 | 15827 | 15827 | 15827 | 15827 | 15827 | 15827 | 15827 |
| ** p<0.05   ** p<0.01   *** p<0.001* | | | | | | | | |

| **Table S17.** GLM Regression against new cases for High 1 cluster from October 1, 2020 to March 1, 2021. The 95% confidence intervals are shown in parenthesis. Estimated coefficients for county-level fixed effects and epidemiological terms (immunity factor and lagged daily cases) are not shown. | | | | | | | | |
| --- | --- | --- | --- | --- | --- | --- | --- | --- |
|  | **All**  **[Eq 1]** | **Absolute Humidity**  **[Eq 2]** | **Retail and Rec**  **[Eq 3]** | **Grocery and Pharmacy**  **[Eq 4]** | **Parks**  **[Eq 5]** | **Transit**  **[Eq 6]** | **Workplaces**  **[Eq 7]** | **Residential**  **[Eq 8]** |
| *Predictors* | *Log-Mean* | *Log-Mean* | *Log-Mean* | *Log-Mean* | *Log-Mean* | *Log-Mean* | *Log-Mean* | *Log-Mean* |
| Intercept | 6.410 ^***^ (6.318 – 6.498) | 6.286 ^***^ (6.196 – 6.374) | 6.400 ^***^ (6.310 – 6.488) | 6.241 ^***^ (6.150 – 6.329) | 6.253 ^***^ (6.162 – 6.341) | 6.285 ^***^ (6.194 – 6.373) | 6.257 ^***^ (6.166 – 6.345) | 6.261 ^***^ (6.171 – 6.349) |
| Absolute Humidity  (14-day Lag) | -0.159 ^***^ (-0.160 – -0.157) | -0.199 ^***^ (-0.200 – -0.198) | -0.208 ^***^ (-0.209 – -0.207) | -0.199 ^***^ (-0.200 – -0.198) | -0.174 ^***^ (-0.175 – -0.173) | -0.199 ^***^ (-0.200 – -0.198) | -0.183 ^***^ (-0.184 – -0.182) | -0.180 ^***^ (-0.181 – -0.179) |
| Retail and Recreation (14-day Lag) | 0.450 ^***^ (0.436 – 0.464) |  | 0.167 ^***^ (0.161 – 0.173) |  |  |  |  |  |
| Grocery Stores and Pharmacies  (14-day Lag) | 0.200 ^***^ (0.191 – 0.209) |  |  | 0.313 ^***^ (0.308 – 0.318) |  |  |  |  |
| Parks  (14-day Lag) | -0.249 ^***^ (-0.254 – -0.243) |  |  |  | -0.188 ^***^ (-0.193 – -0.184) |  |  |  |
| Transit Stations  (14-day Lag) | 0.079 ^***^ (0.068 – 0.089) |  |  |  |  | 0.007  (-0.001 – 0.015) |  |  |
| Workplaces  (14-day Lag) | -0.145 ^***^ (-0.152 – -0.138) |  |  |  |  |  | -0.211 ^***^ (-0.214 – -0.208) |  |
| Residential  (14-day Lag) | 0.218 ^***^ (0.207 – 0.229) |  |  |  |  |  |  | 0.176 ^***^ (0.172 – 0.179) |
| Observations | 10135 | 10135 | 10135 | 10135 | 10135 | 10135 | 10135 | 10135 |
| ** p<0.05   ** p<0.01   *** p<0.001* | | | | | | | | |

| **Table S18.** GLM Regression against new cases for High 2 cluster from October 1, 2020 to March 1, 2021. The 95% confidence intervals are shown in parenthesis. Estimated coefficients for county-level fixed effects and epidemiological terms (immunity factor and lagged daily cases) are not shown. | | | | | | | | |
| --- | --- | --- | --- | --- | --- | --- | --- | --- |
|  | **All**  **[Eq 1]** | **Absolute Humidity**  **[Eq 2]** | **Retail and Rec**  **[Eq 3]** | **Grocery and Pharmacy**  **[Eq 4]** | **Parks**  **[Eq 5]** | **Transit**  **[Eq 6]** | **Workplaces**  **[Eq 7]** | **Residential**  **[Eq 8]** |
| *Predictors* | *Log-Mean* | *Log-Mean* | *Log-Mean* | *Log-Mean* | *Log-Mean* | *Log-Mean* | *Log-Mean* | *Log-Mean* |
| Intercept | 5.188 ^***^ (5.168 – 5.207) | 5.491 ^***^ (5.473 – 5.509) | 5.524 ^***^ (5.506 – 5.542) | 5.446 ^***^ (5.428 – 5.464) | 5.294 ^***^ (5.276 – 5.312) | 5.486 ^***^ (5.468 – 5.504) | 5.413 ^***^ (5.395 – 5.431) | 5.546 ^***^ (5.528 – 5.565) |
| Absolute Humidity  (14-day Lag) | -0.093 ^***^ (-0.093 – -0.092) | -0.127 ^***^ (-0.127 – -0.126) | -0.129 ^***^ (-0.129 – -0.128) | -0.125 ^***^ (-0.126 – -0.125) | -0.114 ^***^ (-0.115 – -0.114) | -0.126 ^***^ (-0.126 – -0.125) | -0.121 ^***^ (-0.122 – -0.121) | -0.118 ^***^ (-0.118 – -0.117) |
| Retail and Recreation (14-day Lag) | 0.511 ^***^ (0.501 – 0.521) |  | 0.089 ^***^ (0.085 – 0.093) |  |  |  |  |  |
| Grocery Stores and Pharmacies  (14-day Lag) | 0.367 ^***^ (0.359 – 0.374) |  |  | 0.294 ^***^ (0.291 – 0.298) |  |  |  |  |
| Parks  (14-day Lag) | -0.736 ^***^ (-0.743 – -0.729) |  |  |  | -0.349 ^***^ (-0.353 – -0.345) |  |  |  |
| Transit Stations  (14-day Lag) | -0.038 ^***^ (-0.046 – -0.031) |  |  |  |  | -0.051 ^***^ (-0.055 – -0.046) |  |  |
| Workplaces  (14-day Lag) | -0.081 ^***^ (-0.086 – -0.077) |  |  |  |  |  | -0.158 ^***^ (-0.160 – -0.156) |  |
| Residential  (14-day Lag) | 0.223 ^***^ (0.216 – 0.230) |  |  |  |  |  |  | 0.187 ^***^ (0.184 – 0.189) |
| Observations | 14456 | 14456 | 14456 | 14456 | 14456 | 14456 | 14456 | 14456 |
| ** p<0.05   ** p<0.01   *** p<0.001* | | | | | | | | |

**Table S19.** The generalized variational inflation factor controlled by degrees of freedom ($GVIF^{1/(2\cdot df)}$) based on the regressions in Tables 1-3 of the main manuscript are shown. The $GVIF^{1/(2\cdot df)}$ values for each variables (column) are shown for each regressions (row).

| **Model [Equation Number]** | **Cluster** | **Absolute Humidity (14-day Lag)** | **Retail and Recreation (14-day Lag)** | **Grocery Stores and Pharmacies (14-day Lag)** | **Parks (14-day Lag)** | **Transit Stations (14-day Lag)** | **Workplaces (14-day Lag)** | **Residential (14-day Lag)** | **Immunity Factor** | **New Cases per 100,000 (14-day Lag)** | **FIPS** |
| --- | --- | --- | --- | --- | --- | --- | --- | --- | --- | --- | --- |
| Absolute Humidity (Entire Year) [Eq 2] | High 1 | 1.38 |  |  |  |  |  |  | 1.48 | 1.10 | 1.00 |
| Absolute Humidity (Entire Year) [Eq 2] | High 2 | 1.60 |  |  |  |  |  |  | 1.53 | 1.20 | 1.01 |
| Absolute Humidity (Entire Year) [Eq 2] | Low 1 | 1.28 |  |  |  |  |  |  | 1.42 | 1.17 | 1.01 |
| Absolute Humidity (Entire Year) [Eq 2] | Low 2 | 1.32 |  |  |  |  |  |  | 1.49 | 1.26 | 1.01 |
| Absolute Humidity (Entire Year) [Eq 2] | Mid 1 | 1.25 |  |  |  |  |  |  | 1.43 | 1.35 | 1.00 |
| Absolute Humidity (Entire Year) [Eq 2] | Mid 2 | 1.44 |  |  |  |  |  |  | 1.44 | 2.11 | 1.01 |
| Absolute Humidity (Mar 2020 - Sep 2020) [Eq 2] | High 1 | 1.96 |  |  |  |  |  |  | 2.07 | 1.31 | 1.01 |
| Absolute Humidity (Mar 2020 - Sep 2020) [Eq 2] | High 2 | 1.57 |  |  |  |  |  |  | 1.48 | 1.21 | 1.01 |
| Absolute Humidity (Mar 2020 - Sep 2020) [Eq 2] | Low 1 | 1.36 |  |  |  |  |  |  | 1.83 | 1.52 | 1.02 |
| Absolute Humidity (Mar 2020 - Sep 2020) [Eq 2] | Low 2 | 1.65 |  |  |  |  |  |  | 1.75 | 1.08 | 1.01 |
| Absolute Humidity (Mar 2020 - Sep 2020) [Eq 2] | Mid 1 | 1.33 |  |  |  |  |  |  | 1.63 | 1.35 | 1.00 |
| Absolute Humidity (Mar 2020 - Sep 2020) [Eq 2] | Mid 2 | 1.31 |  |  |  |  |  |  | 1.65 | 1.24 | 1.01 |
| Absolute Humidity (Oct 2020 - Mar 2021) [Eq 2] | High 1 | 1.64 |  |  |  |  |  |  | 1.75 | 1.06 | 1.01 |
| Absolute Humidity (Oct 2020 - Mar 2021) [Eq 2] | High 2 | 1.61 |  |  |  |  |  |  | 1.60 | 1.12 | 1.01 |
| Absolute Humidity (Oct 2020 - Mar 2021) [Eq 2] | Low 1 | 1.37 |  |  |  |  |  |  | 1.42 | 1.13 | 1.01 |
| Absolute Humidity (Oct 2020 - Mar 2021) [Eq 2] | Low 2 | 1.64 |  |  |  |  |  |  | 1.90 | 1.19 | 1.02 |
| Absolute Humidity (Oct 2020 - Mar 2021) [Eq 2] | Mid 1 | 1.58 |  |  |  |  |  |  | 1.88 | 1.30 | 1.01 |
| Absolute Humidity (Oct 2020 - Mar 2021) [Eq 2] | Mid 2 | 1.56 |  |  |  |  |  |  | 1.72 | 2.06 | 1.01 |
| All (Entire Year) [Eq 1] | High 1 | 1.67 | 3.80 | 2.47 | 2.13 | 3.60 | 3.32 | 4.78 | 1.73 | 1.09 | 1.04 |
| All (Entire Year) [Eq 1] | High 2 | 1.91 | 3.74 | 2.85 | 2.30 | 2.84 | 2.31 | 3.14 | 1.84 | 1.15 | 1.03 |
| All (Entire Year) [Eq 1] | Low 1 | 1.37 | 3.90 | 2.97 | 2.33 | 3.02 | 3.17 | 4.01 | 1.71 | 1.22 | 1.09 |
| All (Entire Year) [Eq 1] | Low 2 | 1.79 | 3.33 | 3.09 | 2.34 | 2.51 | 3.31 | 4.61 | 1.90 | 1.28 | 1.08 |
| All (Entire Year) [Eq 1] | Mid 1 | 1.62 | 3.59 | 2.20 | 2.06 | 3.12 | 3.52 | 5.14 | 1.77 | 1.36 | 1.02 |
| **Model [Equation Number]** | **Cluster** | **Absolute Humidity (14-day Lag)** | **Retail and Recreation (14-day Lag)** | **Grocery Stores and Pharmacies (14-day Lag)** | **Parks (14-day Lag)** | **Transit Stations (14-day Lag)** | **Workplaces (14-day Lag)** | **Residential (14-day Lag)** | **Immunity Factor** | **New Cases per 100,000 (14-day Lag)** | **FIPS** |
| All (Entire Year) [Eq 1] | Mid 2 | 1.81 | 3.61 | 2.30 | 2.09 | 3.34 | 3.47 | 5.08 | 1.79 | 2.20 | 1.04 |
| All (Mar 2020 - Sep 2020) [Eq 1] | High 1 | 2.49 | 4.35 | 2.95 | 2.43 | 4.62 | 4.51 | 6.03 | 2.24 | 1.35 | 1.07 |
| All (Mar 2020 - Sep 2020) [Eq 1] | High 2 | 1.77 | 5.92 | 4.57 | 6.10 | 5.21 | 3.87 | 5.20 | 1.97 | 1.23 | 1.06 |
| All (Mar 2020 - Sep 2020) [Eq 1] | Low 1 | 1.62 | 7.04 | 3.95 | 5.54 | 5.13 | 3.63 | 3.57 | 2.34 | 1.56 | 1.12 |
| All (Mar 2020 - Sep 2020) [Eq 1] | Low 2 | 2.58 | 4.50 | 3.18 | 3.27 | 3.46 | 4.67 | 6.36 | 2.52 | 1.10 | 1.11 |
| All (Mar 2020 - Sep 2020) [Eq 1] | Mid 1 | 1.87 | 6.16 | 2.60 | 2.72 | 5.82 | 8.69 | 10.56 | 2.14 | 1.56 | 1.03 |
| All (Mar 2020 - Sep 2020) [Eq 1] | Mid 2 | 1.92 | 5.12 | 2.40 | 2.65 | 5.20 | 7.45 | 8.39 | 1.86 | 1.29 | 1.04 |
| All (Oct 2020 - Mar 2021) [Eq 1] | High 1 | 1.92 | 4.38 | 3.13 | 2.52 | 5.08 | 3.55 | 5.20 | 1.96 | 1.06 | 1.07 |
| All (Oct 2020 - Mar 2021) [Eq 1] | High 2 | 1.88 | 4.03 | 3.45 | 2.46 | 3.26 | 3.17 | 4.26 | 1.77 | 1.12 | 1.04 |
| All (Oct 2020 - Mar 2021) [Eq 1] | Low 1 | 1.44 | 4.10 | 3.36 | 2.65 | 4.24 | 3.59 | 5.03 | 1.86 | 1.27 | 1.15 |
| All (Oct 2020 - Mar 2021) [Eq 1] | Low 2 | 1.94 | 3.36 | 4.16 | 2.83 | 3.41 | 3.40 | 5.45 | 2.27 | 1.15 | 1.16 |
| All (Oct 2020 - Mar 2021) [Eq 1] | Mid 1 | 1.87 | 3.54 | 2.47 | 2.61 | 3.83 | 3.58 | 5.71 | 2.08 | 1.31 | 1.04 |
| All (Oct 2020 - Mar 2021) [Eq 1] | Mid 2 | 1.85 | 4.25 | 2.96 | 2.65 | 4.47 | 3.69 | 5.85 | 1.92 | 2.03 | 1.06 |
| Grocery and Pharmacy (Entire Year) [Eq 4] | High 1 | 1.37 |  | 1.37 |  |  |  |  | 1.51 | 1.10 | 1.01 |
| Grocery and Pharmacy (Entire Year) [Eq 4] | High 2 | 1.58 |  | 1.36 |  |  |  |  | 1.52 | 1.20 | 1.01 |
| Grocery and Pharmacy (Entire Year) [Eq 4] | Low 1 | 1.30 |  | 1.94 |  |  |  |  | 1.45 | 1.19 | 1.03 |
| Grocery and Pharmacy (Entire Year) [Eq 4] | Low 2 | 1.37 |  | 1.82 |  |  |  |  | 1.50 | 1.27 | 1.03 |
| Grocery and Pharmacy (Entire Year) [Eq 4] | Mid 1 | 1.24 |  | 1.31 |  |  |  |  | 1.48 | 1.36 | 1.01 |
| Grocery and Pharmacy (Entire Year) [Eq 4] | Mid 2 | 1.42 |  | 1.36 |  |  |  |  | 1.51 | 2.10 | 1.01 |
| Grocery and Pharmacy (Mar 2020 - Sep 2020) [Eq 4] | High 1 | 2.11 |  | 1.63 |  |  |  |  | 2.13 | 1.31 | 1.02 |
| Grocery and Pharmacy (Mar 2020 - Sep 2020) [Eq 4] | High 2 | 1.63 |  | 2.63 |  |  |  |  | 1.59 | 1.20 | 1.02 |
| Grocery and Pharmacy (Mar 2020 - Sep 2020) [Eq 4] | Low 1 | 1.37 |  | 1.81 |  |  |  |  | 2.01 | 1.52 | 1.03 |
| Grocery and Pharmacy (Mar 2020 - Sep 2020) [Eq 4] | Low 2 | 1.92 |  | 1.69 |  |  |  |  | 1.75 | 1.08 | 1.03 |
| Grocery and Pharmacy (Mar 2020 - Sep 2020) [Eq 4] | Mid 1 | 1.36 |  | 1.48 |  |  |  |  | 1.77 | 1.41 | 1.01 |
| Grocery and Pharmacy (Mar 2020 - Sep 2020) [Eq 4] | Mid 2 | 1.33 |  | 1.33 |  |  |  |  | 1.80 | 1.24 | 1.01 |
| Grocery and Pharmacy (Oct 2020 - Mar 2021) [Eq 4] | High 1 | 1.57 |  | 1.67 |  |  |  |  | 1.78 | 1.06 | 1.01 |
| Grocery and Pharmacy (Oct 2020 - Mar 2021) [Eq 4] | High 2 | 1.56 |  | 1.65 |  |  |  |  | 1.57 | 1.12 | 1.01 |
| **Model [Equation Number]** | **Cluster** | **Absolute Humidity (14-day Lag)** | **Retail and Recreation (14-day Lag)** | **Grocery Stores and Pharmacies (14-day Lag)** | **Parks (14-day Lag)** | **Transit Stations (14-day Lag)** | **Workplaces (14-day Lag)** | **Residential (14-day Lag)** | **Immunity Factor** | **New Cases per 100,000 (14-day Lag)** | **FIPS** |
| Grocery and Pharmacy (Oct 2020 - Mar 2021) [Eq 4] | Low 1 | 1.37 |  | 2.64 |  |  |  |  | 1.53 | 1.17 | 1.04 |
| Grocery and Pharmacy (Oct 2020 - Mar 2021) [Eq 4] | Low 2 | 1.66 |  | 2.96 |  |  |  |  | 1.89 | 1.18 | 1.05 |
| Grocery and Pharmacy (Oct 2020 - Mar 2021) [Eq 4] | Mid 1 | 1.54 |  | 1.59 |  |  |  |  | 1.86 | 1.32 | 1.01 |
| Grocery and Pharmacy (Oct 2020 - Mar 2021) [Eq 4] | Mid 2 | 1.53 |  | 1.76 |  |  |  |  | 1.75 | 2.02 | 1.02 |
| Parks (Entire Year) [Eq 5] | High 1 | 1.67 |  |  | 1.66 |  |  |  | 1.49 | 1.11 | 1.01 |
| Parks (Entire Year) [Eq 5] | High 2 | 1.79 |  |  | 1.54 |  |  |  | 1.54 | 1.20 | 1.01 |
| Parks (Entire Year) [Eq 5] | Low 1 | 1.35 |  |  | 1.77 |  |  |  | 1.49 | 1.18 | 1.02 |
| Parks (Entire Year) [Eq 5] | Low 2 | 1.75 |  |  | 2.00 |  |  |  | 1.54 | 1.23 | 1.02 |
| Parks (Entire Year) [Eq 5] | Mid 1 | 1.60 |  |  | 1.79 |  |  |  | 1.44 | 1.35 | 1.01 |
| Parks (Entire Year) [Eq 5] | Mid 2 | 1.75 |  |  | 1.78 |  |  |  | 1.44 | 2.12 | 1.01 |
| Parks (Mar 2020 - Sep 2020) [Eq 5] | High 1 | 2.24 |  |  | 2.06 |  |  |  | 2.08 | 1.33 | 1.02 |
| Parks (Mar 2020 - Sep 2020) [Eq 5] | High 2 | 1.56 |  |  | 3.89 |  |  |  | 1.55 | 1.22 | 1.02 |
| Parks (Mar 2020 - Sep 2020) [Eq 5] | Low 1 | 1.36 |  |  | 3.01 |  |  |  | 2.47 | 1.53 | 1.04 |
| Parks (Mar 2020 - Sep 2020) [Eq 5] | Low 2 | 2.16 |  |  | 2.54 |  |  |  | 1.75 | 1.08 | 1.03 |
| Parks (Mar 2020 - Sep 2020) [Eq 5] | Mid 1 | 1.63 |  |  | 2.08 |  |  |  | 1.63 | 1.38 | 1.01 |
| Parks (Mar 2020 - Sep 2020) [Eq 5] | Mid 2 | 1.63 |  |  | 1.99 |  |  |  | 1.65 | 1.24 | 1.01 |
| Parks (Oct 2020 - Mar 2021) [Eq 5] | High 1 | 1.88 |  |  | 2.13 |  |  |  | 1.89 | 1.06 | 1.01 |
| Parks (Oct 2020 - Mar 2021) [Eq 5] | High 2 | 1.80 |  |  | 1.74 |  |  |  | 1.64 | 1.12 | 1.01 |
| Parks (Oct 2020 - Mar 2021) [Eq 5] | Low 1 | 1.39 |  |  | 1.98 |  |  |  | 1.70 | 1.14 | 1.03 |
| Parks (Oct 2020 - Mar 2021) [Eq 5] | Low 2 | 2.02 |  |  | 2.29 |  |  |  | 2.00 | 1.20 | 1.04 |
| Parks (Oct 2020 - Mar 2021) [Eq 5] | Mid 1 | 1.90 |  |  | 1.99 |  |  |  | 1.91 | 1.29 | 1.01 |
| Parks (Oct 2020 - Mar 2021) [Eq 5] | Mid 2 | 1.86 |  |  | 1.99 |  |  |  | 1.76 | 2.07 | 1.02 |
| Residential (Entire Year) [Eq 7] | High 1 | 1.41 |  |  |  |  |  | 1.26 | 1.49 | 1.10 | 1.01 |
| Residential (Entire Year) [Eq 7] | High 2 | 1.61 |  |  |  |  |  | 1.28 | 1.58 | 1.20 | 1.01 |
| Residential (Entire Year) [Eq 7] | Low 1 | 1.30 |  |  |  |  |  | 1.39 | 1.42 | 1.21 | 1.02 |
| Residential (Entire Year) [Eq 7] | Low 2 | 1.37 |  |  |  |  |  | 1.21 | 1.51 | 1.27 | 1.02 |
| Residential (Entire Year) [Eq 7] | Mid 1 | 1.26 |  |  |  |  |  | 1.18 | 1.43 | 1.38 | 1.01 |
| **Model [Equation Number]** | **Cluster** | **Absolute Humidity (14-day Lag)** | **Retail and Recreation (14-day Lag)** | **Grocery Stores and Pharmacies (14-day Lag)** | **Parks (14-day Lag)** | **Transit Stations (14-day Lag)** | **Workplaces (14-day Lag)** | **Residential (14-day Lag)** | **Immunity Factor** | **New Cases per 100,000 (14-day Lag)** | **FIPS** |
| Residential (Entire Year) [Eq 7] | Mid 2 | 1.44 |  |  |  |  |  | 1.26 | 1.45 | 2.13 | 1.01 |
| Residential (Mar 2020 - Sep 2020) [Eq 7] | High 1 | 2.07 |  |  |  |  |  | 1.58 | 2.07 | 1.33 | 1.02 |
| Residential (Mar 2020 - Sep 2020) [Eq 7] | High 2 | 1.55 |  |  |  |  |  | 1.68 | 1.51 | 1.22 | 1.01 |
| Residential (Mar 2020 - Sep 2020) [Eq 7] | Low 1 | 1.40 |  |  |  |  |  | 1.23 | 1.82 | 1.52 | 1.02 |
| Residential (Mar 2020 - Sep 2020) [Eq 7] | Low 2 | 2.09 |  |  |  |  |  | 1.94 | 1.77 | 1.07 | 1.02 |
| Residential (Mar 2020 - Sep 2020) [Eq 7] | Mid 1 | 1.34 |  |  |  |  |  | 1.33 | 1.68 | 1.42 | 1.01 |
| Residential (Mar 2020 - Sep 2020) [Eq 7] | Mid 2 | 1.33 |  |  |  |  |  | 1.27 | 1.67 | 1.24 | 1.01 |
| Residential (Oct 2020 - Mar 2021) [Eq 7] | High 1 | 1.75 |  |  |  |  |  | 1.76 | 1.88 | 1.06 | 1.01 |
| Residential (Oct 2020 - Mar 2021) [Eq 7] | High 2 | 1.74 |  |  |  |  |  | 1.49 | 1.71 | 1.12 | 1.01 |
| Residential (Oct 2020 - Mar 2021) [Eq 7] | Low 1 | 1.41 |  |  |  |  |  | 2.21 | 1.78 | 1.25 | 1.04 |
| Residential (Oct 2020 - Mar 2021) [Eq 7] | Low 2 | 1.74 |  |  |  |  |  | 1.97 | 2.14 | 1.17 | 1.04 |
| Residential (Oct 2020 - Mar 2021) [Eq 7] | Mid 1 | 1.66 |  |  |  |  |  | 1.77 | 1.96 | 1.28 | 1.01 |
| Residential (Oct 2020 - Mar 2021) [Eq 7] | Mid 2 | 1.61 |  |  |  |  |  | 2.04 | 1.85 | 2.12 | 1.02 |
| Retail and Rec (Entire Year) [Eq 3] | High 1 | 1.37 | 1.28 |  |  |  |  |  | 1.45 | 1.10 | 1.01 |
| Retail and Rec (Entire Year) [Eq 3] | High 2 | 1.58 | 1.27 |  |  |  |  |  | 1.52 | 1.20 | 1.01 |
| Retail and Rec (Entire Year) [Eq 3] | Low 1 | 1.31 | 1.73 |  |  |  |  |  | 1.42 | 1.20 | 1.02 |
| Retail and Rec (Entire Year) [Eq 3] | Low 2 | 1.38 | 1.22 |  |  |  |  |  | 1.55 | 1.28 | 1.02 |
| Retail and Rec (Entire Year) [Eq 3] | Mid 1 | 1.24 | 1.16 |  |  |  |  |  | 1.40 | 1.37 | 1.00 |
| Retail and Rec (Entire Year) [Eq 3] | Mid 2 | 1.41 | 1.22 |  |  |  |  |  | 1.42 | 2.12 | 1.01 |
| Retail and Rec (Mar 2020 - Sep 2020) [Eq 3] | High 1 | 2.09 | 1.65 |  |  |  |  |  | 2.07 | 1.32 | 1.02 |
| Retail and Rec (Mar 2020 - Sep 2020) [Eq 3] | High 2 | 1.54 | 2.38 |  |  |  |  |  | 1.50 | 1.22 | 1.01 |
| Retail and Rec (Mar 2020 - Sep 2020) [Eq 3] | Low 1 | 1.35 | 1.93 |  |  |  |  |  | 1.99 | 1.54 | 1.03 |
| Retail and Rec (Mar 2020 - Sep 2020) [Eq 3] | Low 2 | 2.00 | 1.96 |  |  |  |  |  | 1.84 | 1.07 | 1.02 |
| Retail and Rec (Mar 2020 - Sep 2020) [Eq 3] | Mid 1 | 1.36 | 1.26 |  |  |  |  |  | 1.73 | 1.40 | 1.00 |
| Retail and Rec (Mar 2020 - Sep 2020) [Eq 3] | Mid 2 | 1.33 | 1.20 |  |  |  |  |  | 1.70 | 1.24 | 1.01 |
| Retail and Rec (Oct 2020 - Mar 2021) [Eq 3] | High 1 | 1.62 | 1.94 |  |  |  |  |  | 1.73 | 1.06 | 1.01 |
| Retail and Rec (Oct 2020 - Mar 2021) [Eq 3] | High 2 | 1.62 | 1.53 |  |  |  |  |  | 1.59 | 1.12 | 1.01 |
| **Model [Equation Number]** | **Cluster** | **Absolute Humidity (14-day Lag)** | **Retail and Recreation (14-day Lag)** | **Grocery Stores and Pharmacies (14-day Lag)** | **Parks (14-day Lag)** | **Transit Stations (14-day Lag)** | **Workplaces (14-day Lag)** | **Residential (14-day Lag)** | **Immunity Factor** | **New Cases per 100,000 (14-day Lag)** | **FIPS** |
| Retail and Rec (Oct 2020 - Mar 2021) [Eq 3] | Low 1 | 1.38 | 2.58 |  |  |  |  |  | 1.63 | 1.22 | 1.04 |
| Retail and Rec (Oct 2020 - Mar 2021) [Eq 3] | Low 2 | 1.73 | 1.76 |  |  |  |  |  | 1.90 | 1.19 | 1.04 |
| Retail and Rec (Oct 2020 - Mar 2021) [Eq 3] | Mid 1 | 1.68 | 1.74 |  |  |  |  |  | 1.84 | 1.33 | 1.01 |
| Retail and Rec (Oct 2020 - Mar 2021) [Eq 3] | Mid 2 | 1.60 | 2.01 |  |  |  |  |  | 1.77 | 2.08 | 1.02 |
| Transit (Entire Year) [Eq 6] | High 1 | 1.43 |  |  |  | 2.26 |  |  | 1.48 | 1.10 | 1.01 |
| Transit (Entire Year) [Eq 6] | High 2 | 1.61 |  |  |  | 1.79 |  |  | 1.53 | 1.20 | 1.01 |
| Transit (Entire Year) [Eq 6] | Low 1 | 1.29 |  |  |  | 2.07 |  |  | 1.43 | 1.18 | 1.03 |
| Transit (Entire Year) [Eq 6] | Low 2 | 1.38 |  |  |  | 1.61 |  |  | 1.49 | 1.26 | 1.02 |
| Transit (Entire Year) [Eq 6] | Mid 1 | 1.28 |  |  |  | 1.79 |  |  | 1.42 | 1.36 | 1.01 |
| Transit (Entire Year) [Eq 6] | Mid 2 | 1.46 |  |  |  | 1.97 |  |  | 1.44 | 2.13 | 1.02 |
| Transit (Mar 2020 - Sep 2020) [Eq 6] | High 1 | 2.01 |  |  |  | 2.84 |  |  | 2.06 | 1.31 | 1.03 |
| Transit (Mar 2020 - Sep 2020) [Eq 6] | High 2 | 1.55 |  |  |  | 3.06 |  |  | 1.49 | 1.21 | 1.02 |
| Transit (Mar 2020 - Sep 2020) [Eq 6] | Low 1 | 1.36 |  |  |  | 2.17 |  |  | 1.89 | 1.53 | 1.04 |
| Transit (Mar 2020 - Sep 2020) [Eq 6] | Low 2 | 1.90 |  |  |  | 2.21 |  |  | 1.80 | 1.07 | 1.03 |
| Transit (Mar 2020 - Sep 2020) [Eq 6] | Mid 1 | 1.33 |  |  |  | 1.39 |  |  | 1.67 | 1.40 | 1.01 |
| Transit (Mar 2020 - Sep 2020) [Eq 6] | Mid 2 | 1.31 |  |  |  | 1.53 |  |  | 1.69 | 1.24 | 1.01 |
| Transit (Oct 2020 - Mar 2021) [Eq 6] | High 1 | 1.69 |  |  |  | 3.90 |  |  | 1.82 | 1.06 | 1.02 |
| Transit (Oct 2020 - Mar 2021) [Eq 6] | High 2 | 1.68 |  |  |  | 2.52 |  |  | 1.64 | 1.12 | 1.02 |
| Transit (Oct 2020 - Mar 2021) [Eq 6] | Low 1 | 1.38 |  |  |  | 3.20 |  |  | 1.59 | 1.17 | 1.05 |
| Transit (Oct 2020 - Mar 2021) [Eq 6] | Low 2 | 1.70 |  |  |  | 2.55 |  |  | 1.93 | 1.19 | 1.05 |
| Transit (Oct 2020 - Mar 2021) [Eq 6] | Mid 1 | 1.66 |  |  |  | 2.87 |  |  | 1.92 | 1.29 | 1.02 |
| Transit (Oct 2020 - Mar 2021) [Eq 6] | Mid 2 | 1.61 |  |  |  | 3.90 |  |  | 1.81 | 2.09 | 1.03 |
| Workplaces (Entire Year) [Eq 8] | High 1 | 1.42 |  |  |  |  | 1.34 |  | 1.55 | 1.10 | 1.01 |
| Workplaces (Entire Year) [Eq 8] | High 2 | 1.64 |  |  |  |  | 1.22 |  | 1.61 | 1.20 | 1.01 |
| Workplaces (Entire Year) [Eq 8] | Low 1 | 1.29 |  |  |  |  | 1.37 |  | 1.42 | 1.19 | 1.02 |
| Workplaces (Entire Year) [Eq 8] | Low 2 | 1.32 |  |  |  |  | 1.20 |  | 1.53 | 1.27 | 1.02 |
| Workplaces (Entire Year) [Eq 8] | Mid 1 | 1.25 |  |  |  |  | 1.14 |  | 1.43 | 1.37 | 1.00 |
| **Model [Equation Number]** | **Cluster** | **Absolute Humidity (14-day Lag)** | **Retail and Recreation (14-day Lag)** | **Grocery Stores and Pharmacies (14-day Lag)** | **Parks (14-day Lag)** | **Transit Stations (14-day Lag)** | **Workplaces (14-day Lag)** | **Residential (14-day Lag)** | **Immunity Factor** | **New Cases per 100,000 (14-day Lag)** | **FIPS** |
| Workplaces (Entire Year) [Eq 8] | Mid 2 | 1.45 |  |  |  |  | 1.21 |  | 1.46 | 2.14 | 1.01 |
| Workplaces (Mar 2020 - Sep 2020) [Eq 8] | High 1 | 2.02 |  |  |  |  | 1.48 |  | 2.06 | 1.33 | 1.02 |
| Workplaces (Mar 2020 - Sep 2020) [Eq 8] | High 2 | 1.56 |  |  |  |  | 1.46 |  | 1.50 | 1.21 | 1.01 |
| Workplaces (Mar 2020 - Sep 2020) [Eq 8] | Low 1 | 1.38 |  |  |  |  | 1.34 |  | 1.85 | 1.52 | 1.03 |
| Workplaces (Mar 2020 - Sep 2020) [Eq 8] | Low 2 | 1.91 |  |  |  |  | 1.67 |  | 1.74 | 1.07 | 1.02 |
| Workplaces (Mar 2020 - Sep 2020) [Eq 8] | Mid 1 | 1.32 |  |  |  |  | 1.27 |  | 1.73 | 1.41 | 1.00 |
| Workplaces (Mar 2020 - Sep 2020) [Eq 8] | Mid 2 | 1.31 |  |  |  |  | 1.19 |  | 1.70 | 1.24 | 1.01 |
| Workplaces (Oct 2020 - Mar 2021) [Eq 8] | High 1 | 1.71 |  |  |  |  | 1.42 |  | 1.84 | 1.06 | 1.01 |
| Workplaces (Oct 2020 - Mar 2021) [Eq 8] | High 2 | 1.70 |  |  |  |  | 1.21 |  | 1.67 | 1.12 | 1.01 |
| Workplaces (Oct 2020 - Mar 2021) [Eq 8] | Low 1 | 1.38 |  |  |  |  | 1.74 |  | 1.52 | 1.18 | 1.03 |
| Workplaces (Oct 2020 - Mar 2021) [Eq 8] | Low 2 | 1.68 |  |  |  |  | 1.44 |  | 1.96 | 1.18 | 1.03 |
| Workplaces (Oct 2020 - Mar 2021) [Eq 8] | Mid 1 | 1.60 |  |  |  |  | 1.33 |  | 1.91 | 1.28 | 1.01 |

**Table S20.** Levin-Liu-Chu Unit Root Test (stationarity) for all variables included in the panel data regression. The test was conducted for all three periods: entire year (top), Mar 2020 - Sep 2020 (middle), and Oct 2020 - Mar 2021 (bottom). The P-value suggest that all trends are stationary.

| **Duration** | **Variables** | **Lags** | **Observations** | **rho** | **Z-statistic** | **P-value** |
| --- | --- | --- | --- | --- | --- | --- |
| Entire Year | Daily cases per capita | 14 | 112681 | -0.086 | -33.653 | **< 0.001** |
|  | Cumulative cases per capita | 0 | 112695 | -0.013 | -26.934 | **< 0.001** |
|  | Absolute Humidity | 14 | 112681 | -0.005 | -17.869 | **< 0.001** |
|  | Retail and Recreation | 14 | 112681 | -0.022 | -41.975 | **< 0.001** |
|  | Grocery Stores and Pharmacies | 14 | 112681 | -0.025 | -39.175 | **< 0.001** |
|  | Parks | 14 | 112681 | -0.010 | -24.984 | **< 0.001** |
|  | Transit Stations | 14 | 112681 | -0.012 | -28.633 | **< 0.001** |
|  | Workplaces | 14 | 112681 | -0.037 | -47.159 | **< 0.001** |
|  | Residential | 14 | 112681 | -0.025 | -42.052 | **< 0.001** |
| Mar 2020 - Sep 2020 | Daily cases per capita | 8 | 45445 | -0.099 | -26.592 | **< 0.001** |
|  | Cumulative cases per capita | 0 | 45453 | -0.020 | -21.581 | **< 0.001** |
|  | Absolute Humidity | 1 | 45452 | -0.015 | -19.328 | **< 0.001** |
|  | Retail and Recreation | 7 | 45446 | -0.031 | -33.179 | **< 0.001** |
|  | Grocery Stores and Pharmacies | 10 | 45443 | -0.032 | -30.413 | **< 0.001** |
|  | Parks | 14 | 45439 | -0.017 | -18.978 | **< 0.001** |
|  | Transit Stations | 10 | 45443 | -0.021 | -24.641 | **< 0.001** |
|  | Workplaces | 14 | 45439 | -0.062 | -39.066 | **< 0.001** |
|  | Residential | 14 | 45439 | -0.039 | -33.346 | **< 0.001** |
| Oct 2020 - Mar 2021 | Daily cases per capita | 14 | 67227 | -0.110 | -28.846 | **< 0.001** |
|  | Cumulative cases per capita | 0 | 67241 | -0.016 | -23.293 | **< 0.001** |
|  | Absolute Humidity | 14 | 67227 | -0.011 | -18.176 | **< 0.001** |
|  | Retail and Recreation | 14 | 67227 | -0.018 | -23.133 | **< 0.001** |
|  | Grocery Stores and Pharmacies | 14 | 67227 | -0.019 | -22.313 | **< 0.001** |
|  | Parks | 14 | 67227 | -0.019 | -25.222 | **< 0.001** |
|  | Transit Stations | 14 | 67227 | -0.011 | -18.920 | **< 0.001** |
|  | Workplaces | 14 | 67227 | -0.026 | -26.548 | **< 0.001** |
|  | Residential | 14 | 67227 | -0.016 | -20.759 | **< 0.001** |

**Table S21.** McFadden R^2^ measure for all GLMs.

| **Model** | **Low 1** | **Low 2** | **Mid 1** | **Mid 2** | **High 1** | **High 2** |
| --- | --- | --- | --- | --- | --- | --- |
| All (Entire Year) [Eq 1] | 0.49 | 0.40 | 0.47 | 0.53 | 0.38 | 0.39 |
| All (Mar 2020 - Sep 2020) [Eq 1] | 0.53 | 0.34 | 0.44 | 0.40 | 0.49 | 0.50 |
| All (Oct 2020 - Mar 2021) [Eq 1] | 0.51 | 0.49 | 0.47 | 0.58 | 0.40 | 0.49 |
| Absolute Humidity (Entire Year) [Eq 2] | 0.42 | 0.33 | 0.39 | 0.47 | 0.26 | 0.29 |
| Absolute Humidity (Mar 2020 - Sep 2020) [Eq 2] | 0.46 | 0.27 | 0.37 | 0.34 | 0.43 | 0.45 |
| Absolute Humidity (Oct 2020 - Mar 2021) [Eq 2] | 0.44 | 0.40 | 0.37 | 0.51 | 0.34 | 0.41 |
| Retail and Rec (Entire Year) [Eq 3] | 0.44 | 0.35 | 0.40 | 0.47 | 0.28 | 0.30 |
| Retail and Rec (Mar 2020 - Sep 2020) [Eq 3] | 0.47 | 0.29 | 0.38 | 0.34 | 0.43 | 0.48 |
| Retail and Rec (Oct 2020 - Mar 2021) [Eq 3] | 0.45 | 0.41 | 0.37 | 0.51 | 0.34 | 0.42 |
| Grocery and Pharmacy (Entire Year) [Eq 4] | 0.43 | 0.33 | 0.40 | 0.47 | 0.27 | 0.29 |
| Grocery and Pharmacy (Mar 2020 - Sep 2020) [Eq 4] | 0.46 | 0.28 | 0.38 | 0.35 | 0.44 | 0.48 |
| Grocery and Pharmacy (Oct 2020 - Mar 2021) [Eq 4] | 0.45 | 0.43 | 0.42 | 0.53 | 0.36 | 0.43 |
| Parks (Entire Year) [Eq 5] | 0.42 | 0.33 | 0.39 | 0.47 | 0.27 | 0.29 |
| Parks (Mar 2020 - Sep 2020) [Eq 5] | 0.46 | 0.27 | 0.37 | 0.34 | 0.43 | 0.49 |
| Parks (Oct 2020 - Mar 2021) [Eq 5] | 0.47 | 0.40 | 0.37 | 0.51 | 0.35 | 0.43 |
| Transit (Entire Year) [Eq 6] | 0.43 | 0.33 | 0.39 | 0.47 | 0.26 | 0.29 |
| Transit (Mar 2020 - Sep 2020) [Eq 6] | 0.46 | 0.31 | 0.37 | 0.34 | 0.44 | 0.47 |
| Transit (Oct 2020 - Mar 2021) [Eq 6] | 0.45 | 0.40 | 0.37 | 0.51 | 0.34 | 0.41 |
| Workplaces (Entire Year) [Eq 7] | 0.43 | 0.33 | 0.39 | 0.47 | 0.27 | 0.29 |
| Workplaces (Mar 2020 - Sep 2020) [Eq 7] | 0.46 | 0.30 | 0.37 | 0.34 | 0.44 | 0.47 |
| Workplaces (Oct 2020 - Mar 2021) [Eq 7] | 0.46 | 0.41 | 0.39 | 0.53 | 0.36 | 0.43 |
| Residential (Entire Year) [Eq 8] | 0.44 | 0.34 | 0.39 | 0.47 | 0.27 | 0.29 |
| Residential (Mar 2020 - Sep 2020) [Eq 8] | 0.47 | 0.29 | 0.37 | 0.34 | 0.44 | 0.47 |
| Residential (Oct 2020 - Mar 2021) [Eq 8] | 0.47 | 0.41 | 0.39 | 0.53 | 0.35 | 0.42 |

**Table S22.** The average error quantified as Mean Square Error (MSE) for 100 iterations using K-fold cross-validation.

|  | | | | | | | |
| --- | --- | --- | --- | --- | --- | --- | --- |
| **Period** | **Model^*^** | **Low 1** | **Low 2** | **Mid 1** | **Mid 2** | **High 1** | **High 2** |
| Entire Year | All Variables [Eq 1] | 57769.6 | 36392.6 | 256334.8 | 528720.0 | 33249.7 | 154522.9 |
|  | Humidity [Eq 2] | 57774.7 | 36398.1 | 256345.1 | 528728.5 | 33258.3 | 154533.6 |
|  | Retail and Rec [Eq 3] | 57773.3 | 36396.2 | 256343.7 | 528727.7 | 33256.8 | 154532.3 |
|  | Grocery and Pharmacy [Eq 4] | 57774.6 | 36398.0 | 256343.8 | 528726.7 | 33257.4 | 154532.9 |
|  | Parks [Eq 5] | 57774.3 | 36398.2 | 256345.0 | 528728.0 | 33258.3 | 154533.4 |
|  | Transit [Eq 6] | 57774.4 | 36398.2 | 256344.9 | 528728.5 | 33258.2 | 154533.6 |
|  | Workplaces [Eq 7] | 57774.2 | 36398.0 | 256345.1 | 528728.5 | 33258.2 | 154533.5 |
|  | Residential [Eq 8] | 57773.2 | 36397.3 | 256345.1 | 528728.1 | 33257.9 | 154533.6 |
| March 2020 to September 2020 | All Variables [Eq 1] | 14240.4 | 5806.2 | 69901.1 | 87476.9 | 7501.8 | 99234.2 |
|  | Humidity [Eq 2] | 14244.0 | 5807.1 | 69906.6 | 87483.4 | 7503.0 | 99240.3 |
|  | Retail and Rec [Eq 3] | 14244.0 | 5806.7 | 69906.2 | 87482.6 | 7503.0 | 99237.5 |
|  | Grocery and Pharmacy [Eq 4] | 14243.9 | 5807.1 | 69905.6 | 87480.8 | 7503.0 | 99236.4 |
|  | Parks [Eq 5] | 14242.5 | 5807.0 | 69906.0 | 87482.9 | 7503.0 | 99239.2 |
|  | Transit [Eq 6] | 14243.7 | 5806.6 | 69906.6 | 87483.0 | 7502.8 | 99240.0 |
|  | Workplaces [Eq 7] | 14244.0 | 5806.5 | 69906.5 | 87482.5 | 7502.7 | 99239.6 |
|  | Residential [Eq 8] | 14244.0 | 5806.6 | 69906.5 | 87482.7 | 7502.8 | 99240.0 |
| October 2020 to March 2021 | All Variables [Eq 1] | 49617.5 | 198298.9 | 84056.9 | 53860.3 | 371812.3 | 842321.6 |
|  | Humidity [Eq 2] | 49628.0 | 198309.5 | 84062.3 | 53861.3 | 371823.7 | 842331.8 |
|  | Retail and Rec [Eq 3] | 49624.4 | 198309.5 | 84062.3 | 53860.9 | 371823.3 | 842331.7 |
|  | Grocery and Pharmacy [Eq 4] | 49621.1 | 198308.6 | 84062.2 | 53861.0 | 371819.0 | 842328.1 |
|  | Parks [Eq 5] | 49628.0 | 198306.8 | 84060.3 | 53861.3 | 371823.6 | 842331.4 |
|  | Transit [Eq 6] | 49625.3 | 198309.5 | 84062.0 | 53861.3 | 371823.7 | 842331.4 |
|  | Workplaces [Eq 7] | 49627.9 | 198308.3 | 84062.4 | 53861.3 | 371821.4 | 842331.9 |
|  | Residential [Eq 8] | 49627.1 | 198308.4 | 84062.3 | 53861.3 | 371822.4 | 842331.9 |

^*^The county-level fixed effects were removed from our cross-validation analysis.

|  |  |  |  |  |  |  |  |
| --- | --- | --- | --- | --- | --- | --- | --- |
| **Period** | **Variables** | **Low 1** | **Low 2** | **Mid 1** | **Mid 2** | **High 1** | **High 2** |
| Entire Year | (Intercept) | (3.745 - 3.75) | (3.341 - 3.344) | (3.768 - 3.77) | (3.874 - 3.876) | (3.501 - 3.502) | (2.84 - 2.842) |
|  | Immunity Factor | (-2.061 - -2.044) | (2.571 - 2.618) | (0.729 - 0.775) | (2.528 - 2.549) | (6.01 - 6.025) | (5.178 - 5.191) |
|  | Grocery Stores and Pharmacies (14-day Lag) | (-0.158 - -0.157) | (-0.015 - -0.014) | (0.09 - 0.091) | (0.277 - 0.278) | (0.108 - 0.109) | (0.039 - 0.04) |
|  | Absolute Humidity (14-day Lag) | (-0.177 - -0.176) | (-0.052 - -0.051) | (-0.098 - -0.098) | (-0.12 - -0.119) | (-0.055 - -0.055) | (-0.004 - -0.004) |
|  | New Cases per 100,000 (14-day Lag) | (0.011 - 0.011) | (0.007 - 0.007) | (0.006 - 0.006) | (0.007 - 0.007) | (0.002 - 0.002) | (0.005 - 0.005) |
|  | Parks (14-day Lag) | (-0.232 - -0.232) | (-0.047 - -0.046) | (-0.122 - -0.121) | (-0.232 - -0.231) | (-0.15 - -0.15) | (-0.587 - -0.586) |
|  | Residential (14-day Lag) | (-0.233 - -0.232) | (-0.319 - -0.316) | (-0.002 - 0.001) | (-0.214 - -0.213) | (-0.369 - -0.368) | (-0.033 - -0.032) |
|  | Retail and Recreation (14-day Lag) | (0.409 - 0.411) | (0.442 - 0.444) | (0.569 - 0.571) | (0.201 - 0.202) | (0.469 - 0.47) | (0.826 - 0.827) |
|  | Transit Stations (14-day Lag) | (0.022 - 0.023) | (-0.357 - -0.355) | (-0.241 - -0.241) | (-0.138 - -0.137) | (-0.053 - -0.052) | (-0.121 - -0.12) |
|  | Workplaces (14-day Lag) | (-0.22 - -0.22) | (-0.309 - -0.307) | (-0.249 - -0.248) | (-0.331 - -0.33) | (-0.564 - -0.563) | (-0.328 - -0.327) |
|  | ***Mean Square Error (MSE)*** | ***(51443 - 64096)*** | ***(32838 - 39947)*** | ***(228689 - 283981)*** | ***(419056 - 638384)*** | ***(30715 - 35785)*** | ***(133591 - 175455)*** |
| March 2020 to September 2020 | (Intercept) | (2.018 - 2.021) | (1.898 - 1.901) | (2.787 - 2.789) | (2.866 - 2.869) | (1.544 - 1.546) | (-0.183 - -0.178) |
|  | Immunity Factor | (8.208 - 8.336) | (45.452 - 46.484) | (11.399 - 11.48) | (27.059 - 27.183) | (1.117 - 1.208) | (5.257 - 5.382) |
|  | Grocery Stores and Pharmacies (14-day Lag) | (0.017 - 0.018) | (0.144 - 0.146) | (0.3 - 0.301) | (0.421 - 0.422) | (0.097 - 0.098) | (0.329 - 0.331) |
|  | Absolute Humidity (14-day Lag) | (-0.012 - -0.011) | (-0.022 - -0.022) | (-0.064 - -0.064) | (-0.076 - -0.076) | (0.035 - 0.035) | (0.16 - 0.161) |
|  | New Cases per 100,000 (14-day Lag) | (0.024 - 0.024) | (0.007 - 0.008) | (0.017 - 0.017) | (0.013 - 0.013) | (0.025 - 0.025) | (0.006 - 0.007) |
|  | Parks (14-day Lag) | (-0.329 - -0.328) | (0.006 - 0.006) | (-0.16 - -0.159) | (-0.234 - -0.233) | (-0.044 - -0.043) | (-0.226 - -0.224) |
|  | Residential (14-day Lag) | (-0.053 - -0.051) | (-0.107 - -0.102) | (0.133 - 0.134) | (-0.002 - -0.001) | (-0.061 - -0.059) | (0.147 - 0.15) |
|  | Retail and Recreation (14-day Lag) | (0.606 - 0.61) | (-0.033 - -0.031) | (0.394 - 0.396) | (0.086 - 0.087) | (0.344 - 0.346) | (0.389 - 0.392) |
|  | Transit Stations (14-day Lag) | (-0.165 - -0.163) | (-0.107 - -0.105) | (-0.259 - -0.258) | (-0.082 - -0.08) | (-0.085 - -0.085) | (-0.156 - -0.155) |
|  | Workplaces (14-day Lag) | (-0.278 - -0.276) | (-0.199 - -0.196) | (-0.118 - -0.116) | (-0.126 - -0.124) | (-0.333 - -0.332) | (-0.045 - -0.042) |
|  | ***Mean Square Error (MSE)*** | ***(11531 - 16949)*** | ***(5164 - 6448)*** | ***(59859 - 79943)*** | ***(72873 - 102081)*** | ***(6943 - 8060)*** | ***(63483 - 134986)*** |
| October 2020 to March 2021 | (Intercept) | (4.025 - 4.03) | (3.631 - 3.635) | (4.289 - 4.292) | (4.511 - 4.513) | (4.952 - 4.954) | (3.712 - 3.716) |
|  | Immunity Factor | (-10.699 - -10.68) | (-3.087 - -3.046) | (-4.962 - -4.924) | (-3.65 - -3.626) | (-6.963 - -6.948) | (-0.03 - -0.015) |
|  | Grocery Stores and Pharmacies (14-day Lag) | (0.117 - 0.118) | (0.066 - 0.067) | (0.325 - 0.326) | (0.537 - 0.538) | (0.435 - 0.436) | (0.402 - 0.403) |
|  | Absolute Humidity (14-day Lag) | (-0.018 - -0.017) | (0.012 - 0.013) | (-0.067 - -0.067) | (-0.132 - -0.132) | (-0.102 - -0.102) | (-0.03 - -0.03) |
|  | New Cases per 100,000 (14-day Lag) | (0.008 - 0.008) | (0.006 - 0.006) | (0.006 - 0.006) | (0.007 - 0.007) | (0.002 - 0.002) | (0.004 - 0.004) |
|  | Parks (14-day Lag) | (-0.424 - -0.423) | (-0.047 - -0.046) | (-0.103 - -0.102) | (-0.179 - -0.177) | (-0.189 - -0.188) | (-0.555 - -0.554) |
|  | Residential (14-day Lag) | (0.202 - 0.204) | (-0.088 - -0.085) | (0.168 - 0.17) | (0.049 - 0.051) | (-0.048 - -0.047) | (0.151 - 0.152) |
|  | Retail and Recreation (14-day Lag) | (0.252 - 0.253) | (0.174 - 0.176) | (0.206 - 0.207) | (-0.211 - -0.21) | (0.128 - 0.129) | (0.211 - 0.212) |
|  | Transit Stations (14-day Lag) | (0.164 - 0.165) | (-0.096 - -0.094) | (0.024 - 0.025) | (0.031 - 0.032) | (0.115 - 0.116) | (-0.013 - -0.012) |
|  | Workplaces (14-day Lag) | (-0.035 - -0.034) | (-0.14 - -0.138) | (-0.135 - -0.134) | (-0.014 - -0.012) | (-0.223 - -0.222) | (-0.082 - -0.081) |
|  | ***Mean Square Error (MSE)*** | ***(72598 - 95516)*** | ***(48138 - 59583)*** | ***(330648 - 412977)*** | ***(665559 - 1019084)*** | ***(45538- 53697)*** | ***(180151 - 216440)*** |

**Table S23**. The 95% confidence intervals of parameter estimation from K-fold cross-validation analysis of GLM with all variables (Absolute Humidity + All Google Mobility Trends) [Eq 1]. The cross-validation was conducted over 100 folds. Compared to the single regression, all parameter estimations are consistent and robust.


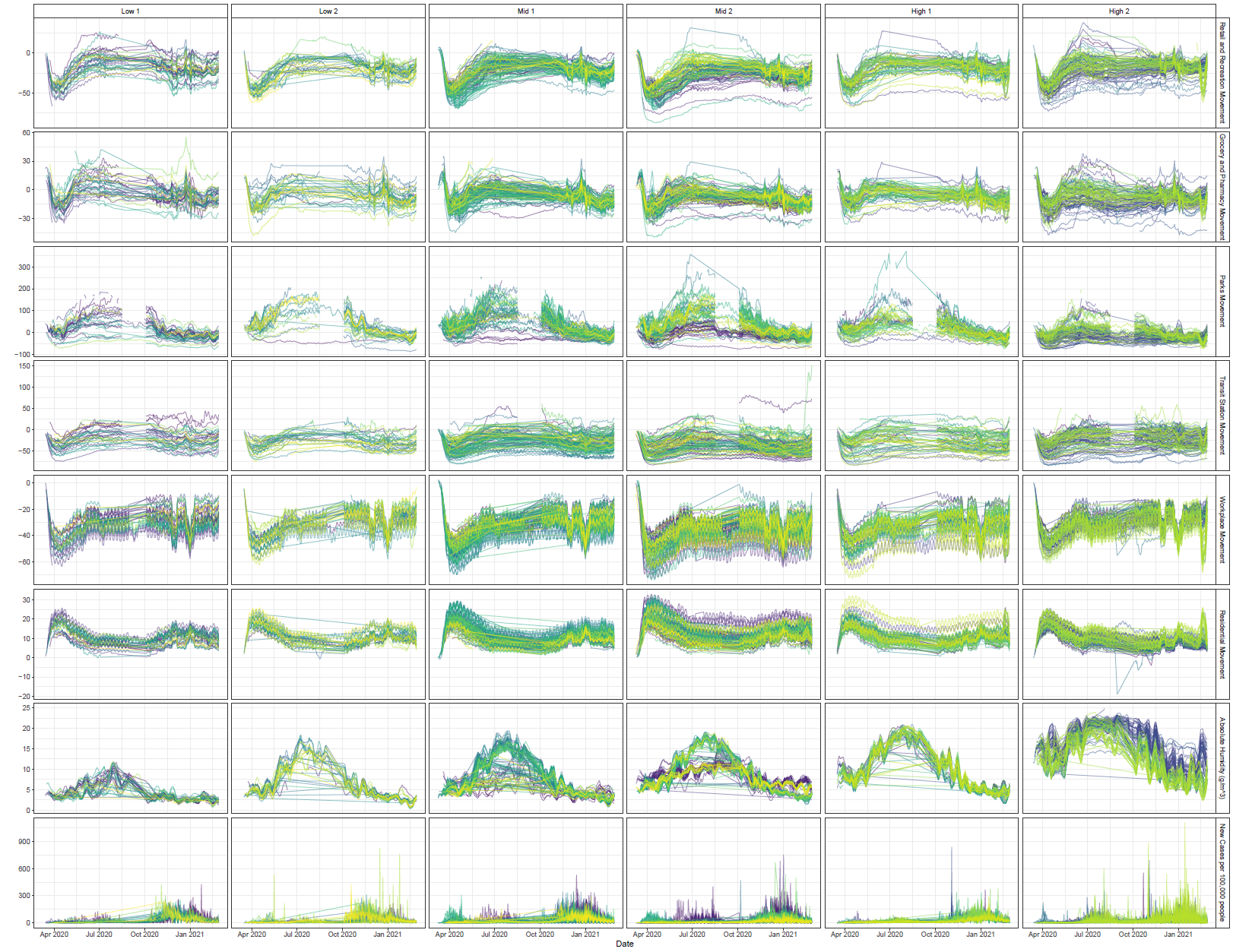


**Figure S1. Google mobility measures, absolute humidity, and daily case trends based on absolute humidity clusters.** All three series are based on a smoothed 7-day moving average.


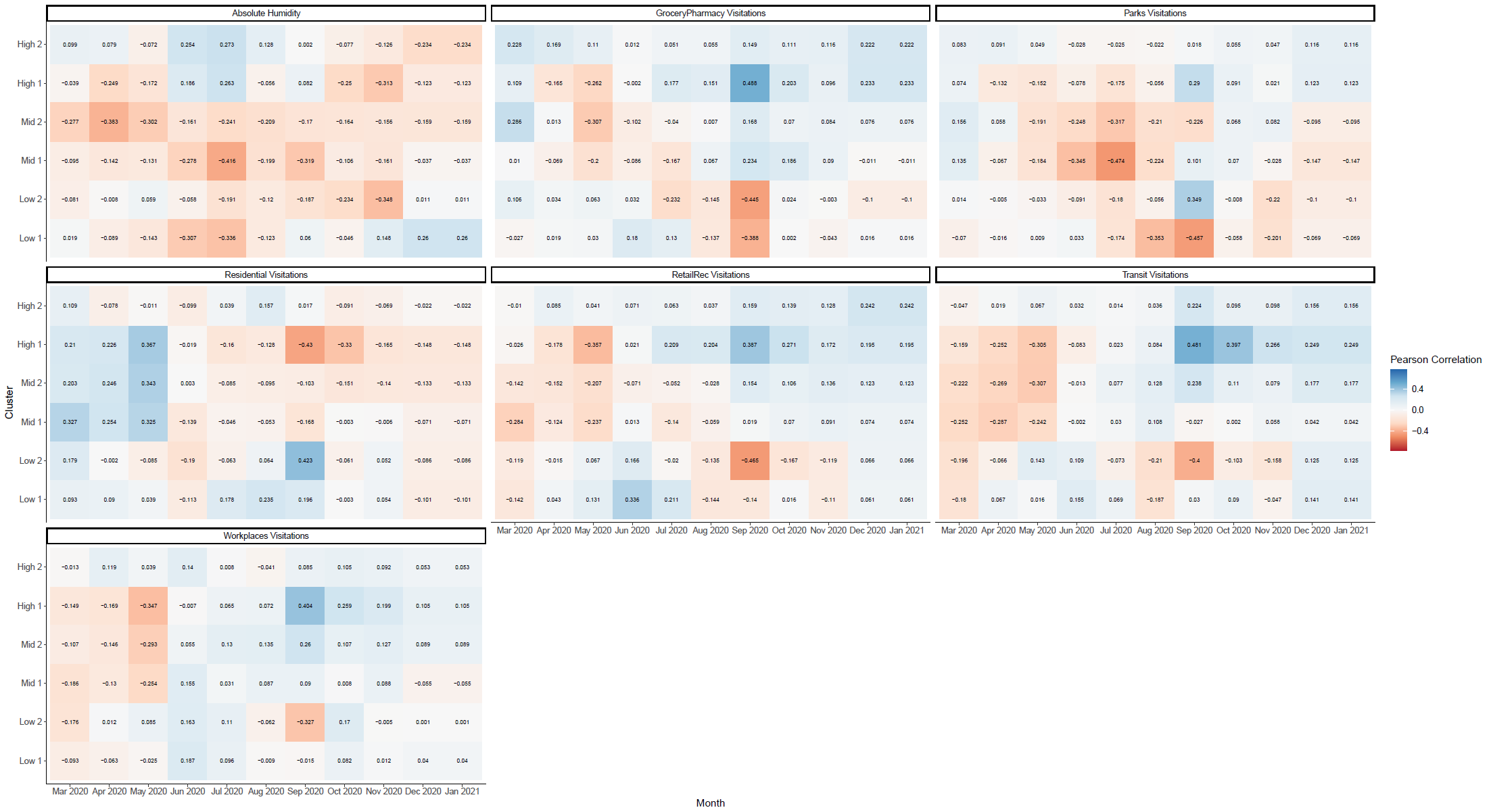


**Figure S2.** Pearson correlation measure for 14-day lagged measures of smoothed daily absolute humidity and Google mobility measures against new cases in each respective humidity cluster and month.
